# Humanized monoacylglycerol acyltransferase 2 mice develop metabolic dysfunction-associated steatohepatitis

**DOI:** 10.1016/j.jlr.2024.100695

**Published:** 2024-11-05

**Authors:** J. Jose Corbalan, Pranavi Jagadeesan, Karla K. Frietze, Rulaiha Taylor, Grace L. Gao, Grant Gallagher, Joseph T. Nickels

**Affiliations:** 1 The Institute of Metabolic Disorders, Genesis Research and Development Institute, Genesis Biotechnology Group, Hamilton, NJ, USA; 2 Department of Pharmacology and Toxicology, Earnest Mario School of Pharmacy, Rutgers University, Piscataway, NJ, USA; 3 Rutgers Center for Lipid Research, New Jersey Institute for Food, Nutrition, and Health, Rutgers University, New Brunswick, NJ, USA; 4 Oncoveda, Genesis Research and Development Institute, Genesis Biotechnology Group, Hamilton, NJ, USA

**Keywords:** lipids, MASH, MASLD, monoacylglycerol acyltransferase, bile acids, RNASeq

## Abstract

Mice lacking monoacylglycerol acyltransferase 2 (mMGAT2^1^) are resistant to diet-induced fatty liver, suggesting hMOGAT2 inhibition is a viable option for treating metabolic dysfunction-associated steatotic liver disease (MASLD)/metabolic dysfunction-associated steatohepatitis (MASH). We generated humanized *hMOGAT2* mice (*HuMgat2*) for use in pre-clinical studies testing the efficacy of hMOGAT2 inhibitors for treating MASLD/MASH. *HuMgat2* mice developed MASH when fed a steatotic diet. Computer-aided histology revealed the presence of hepatocyte cell ballooning, immune cell infiltration, and fibrosis. Hepatocytes accumulated Mallory-Denk bodies containing phosphorylated p62/sequestosome-1-ubiquitinated protein aggregates likely caused by defects in autophagy. Metainflammation and apoptotic cell death were seen in the livers of *HuMgat2* mice. Treating *HuMgat2* mice with elafibranor reduced several MASH phenotypes. RNASeq analysis predicted changes in bile acid transporter expression that correlated with altered bile acid metabolism indicative of cholestasis. Our results suggest that *HuMgat2* mice will serve as a pre-clinical model for testing hMOGAT2 inhibitor efficacy and toxicity and allow for the study of hMOGAT2 in the context of MASH.

## Graphical abstract

**Figure:**
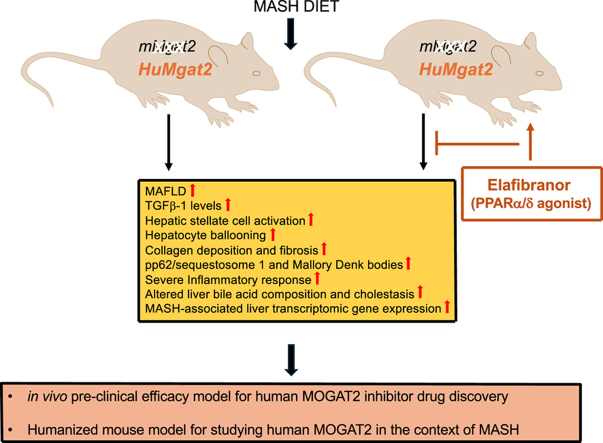

It is estimated that between 20% and 45% of individuals in the United States have MASLD (1). Presently, there are no diagnostic tests and only one FDA-approved drug (Rezdiffra) to help physicians recognize and treat the disease, resulting in a huge unmet medical need for diagnostics and additional drug therapies (2).

A link exists between increased accumulation of liver triglycerides and severe forms of MASLD, which include MASH, fibrosis, and cirrhosis (3, 4). Triglycerides in the liver are synthesized mainly by the glycerol 3-phosphate pathway (5). Lesser amounts are produced by the monoacylglycerol pathway (MGAT2 and diacylglycerol acyltransferase (DGAT1/2) using triglyceride hydrolysis products generated by triglycerides lipases (6). Triglycerides can also come from the diet. These triglycerides are hydrolyzed in the intestinal lumen to monoacylglycerides and fatty acids by pancreatic lipase where they are transported into intestinal enterocytes and used for triglycerides resynthesis by MGAT2 and DGAT1 (7). Resynthesized triglycerides are associated with chylomicrons that enter the lymph system and are transported to the liver (8). Dietary-derived obesity and insulin resistance brought on by caloric overload are known risk factors for developing MASLD/MASH (9).

MGAT1/2/3 makes up the monoacylglycerol acyltransferase family in rodents and humans (7). All three are related to the DGAT2 diacylglycerol acyltransferase family (6). *hMOGAT1* expression is highest in the liver and kidney (10), whereas *mMGAT1* mRNA has been detected in the stomach and kidney (10). *MGAT3* is not expressed in mice but is found in humans and higher mammals, where it is expressed in the intestines and pancreas (11, 12).

hMOGAT2 protein sequence shares 81% and 45% homologies with mMGAT2 and mDGAT2, respectively, whereas both hMGAT1/3 and mMGAT1/3 lack any significant conservation with the sequence (13, 14). *hMOGAT2* mRNA has been detected in human small intestines, liver, stomach, adipose, kidney, and colon (15). *mMGAT2* expression is found in the duodenum of the small intestines, where the highest level of enzyme activity has been observed (13). *h*MOGAT2 enzyme activity is highest in the small intestine, while low levels have been detected in the liver, kidney, and lung (13).

*mMgat2* mice are resistant to high-fat diet-induced obesity, insulin resistance, and hepatic steatosis (6, 16), phenotypes that are restored when *hMOGAT2* is expressed in the intestines of these mice (17), pointing to intestinal MGAT2 playing a major role in regulating whole-body triglyceride homeostasis in mice, and possibly humans.

Several hMOGAT2 inhibitors have been shown to reduce hepatic triglyceride levels and hepatic steatosis in mice fed multiple MASH diets (18, 19, 20). Cheng *et al.*, (21) have shown that the hMOGAT2 inhibitor BMS-963272 attenuates fibrosis progression in mice fed two different MASH diets. The HuR RNA-binding protein has been shown to bind to intron 1 of *MGAT2* pre-mRNA resulting in its stabilization (22). Loss of intestinal HuR reduces MASLD and obesity (22). Currently, a hMOGAT inhibitor has finished phase II clinical trials for weight loss, and efficacy data may be released sometime in 2024 [NCT0592514]. As obesity is a major risk factor for developing MASLD/MASH, and weight modulates the severity of MASH (23), the results of the trial will be of great interest.

The widespread use of orally available drugs is the easiest and most optimal treatment. It reduces noncompliance issues arising from using more invasive methods of drug delivery, like intravenous, intramuscular, and subcutaneous injections. Unfortunately, systemic side effects due to high oral bioavailability may not be observed in pre-clinical studies but arise only during human clinical trials. There are many publications documenting these cases (24, 25). The use of humanized murine models in pre-clinical studies could reveal human-target drug interaction effects before any extensive medicinal chemistry and clinical trial efforts are put forth at a huge expense. These models have the additional benefit of being used as a physiologically relevant in vivo SAR tool for target optimization, and for studying the human gene in the context of a particular disease.

We generated *HuMgat2* mice expressing *hMOGAT2* in place of *mMgat2* to use in pre-clinical hMOGAT2 inhibitor toxicity studies. We determined how *HuMgat2* mice responded to being fed a MASH diet and their therapeutic response to the PPARα/δ dual agonist, elafibranor, a phase III clinical trial drug tested for treating MASH (26, 27, 28). *HuMgat2* mice fed a CDAA-HFD developed MASH with fibrosis. Hepatocyte apoptotic cell death was observed with macrophage infiltration leading to an ongoing metainflammatory cytokine response. Treating *HuMgat2* mice with elafibranor reduced the severity of several phenotypes associated with MASLD/MASH. RNASeq analysis predicted that *HuMgat2* mice with MASH had changes in bile acid transporter expression that would cause the accumulation of toxic bile acids resulting in cholestasis. Our results suggest that *HuMgat2* mice are a valid model useful in elucidating the pharmacological and physiological properties of MOGAT2 inhibitors.

## Materials and methods

### Generation and validation of *HuMgat2* mice

*HuMgat2* mice were generated in the C57BL/6J background by replacing the endogenous *mMgat2* locus with a *hMOGAT2* full-length cDNA coding sequence, whose expression was driven by the endogenous *mMgat2* promoter, resulting in whole-body protein expression of hMOGAT2 in place of endogenous mMGAT2.

*HuMgat2* knock-in mice were commissioned from Cyagen (Santa Clara, CA) (*supplemental Cyagen HuMgat2 generation design*). Briefly, the human *MOGAT2* coding sequence NM_025098.3, including the start and stop codons, was used to replace the endogenous sequence on chromosome 7 of C57 Bl/6 ES cells, by LoxP/Cre-mediated recombination. Recombinant cells were selected using neomycin resistance. Selected cells were injected into 3-day-old blastocysts and transplanted into pseudopregnant recipient females. Fully transgenic animals were selected via cross-breeding of first-generation chimeras via PCR screening. *HuMgat2* mice were used at 4–10 weeks of age. The Invivotek institutional animal care and use committee approved all animal studies. All studies followed procedures according to the “*Institutional Animal Care and Use Committee Handbook*”.

The levels of human MOGAT2 and mouse MGAT2 protein levels in liver and small intestine tissues from *mMgat2* and *HuMgat2* mice were determined using anti-human MGAT2 antibodies (Abcam, Waltham, MA., #ab228950) and anti-mouse MGAT2 antibodies (Millipore Sigma Aldrich, SAB 1300155) (supplemental Fig. S1A). The levels of *HuMgat2* MOGAT2 protein in livers and small intestines were elevated 1.36- and 1.26-fold compared to *mMgat2* MGAT2 levels, respectively (supplemental Fig. S1B). *q*RT-PCR analysis determined there were no differences in the levels of gene expression of human and mouse *MGAT2* (supplemental Fig. S1C).

### Antibodies

Anti-Ub antibodies (Cat# 58395), anti-COL1A1 antibodies (Cat# 72026), anti-αSMA antibodies (Cat# 14968), anti-PARP1 antibodies (Cat# 9542), and anti-GAPDH antibodies (Cat# 2118) were from Cell Signaling Technology and used at a 1:1000 dilution. Anti-p62 antibodies (Cat# sc-28359) was from Santa Cruz Biotechnology and used at a 1:1,500 dilution. anti-pp62^Ser403^ antibodies (Cat# ab124762) were from Abcam and used at a 1:2,000 dilution. Anti-COL3A1 antibodies (Cat# PA5-34787) and anti-caspase 3 antibodies (Cat# MA1-91637) were from ThermoFisher Scientific and used at 1:1,000 and 1:15,000, respectively.

### Miscellaneous reagents

The Ubiquitin enrichment kit (Pierce™) was from ThermoFisher Scientific (Cat# PI89899). AI-aided histology algorithm software (Reveal Biosciences, https://www.revealbio.com/nash/).

### Metabolic feeding studies

Six weeks old male *mMgat2* and *HuMgat2* mice were maintained on a 12-h light/dark cycle with food and water fed ad libitum. Mice were initially maintained on a regular chow diet for 2 weeks for acclimation to the facility (Purina, Picochow 5053, Lab Diets). Mice were then divided into 3 cohorts. Overall, the mice were fed the CDAA-HFD for a total of 24 weeks (#A06071302, Research Diets, New Brunswick, NJ). The first 8 weeks all cohorts were fed the CDAA-HFD only. At week 8 one *mMgat2* and one *HuMgat2* cohort was given elafibranor for 16 weeks (n = 8). The data collected are from weeks 8–24. Body weights and food intake were measured weekly. We point out that it has been published that mice fed the CDAA-HFD do not gain weight (21, 29, 30). Body weights were analyzed by a two-way ANOVA with genotype as a between-group factor and the time course as a within-subject factor (repeated measures).

### Serum clinical chemistries

Serum was collected from *mMgat2* and *HuMgat2* mice by a cardiac bleed after 24 weeks of feeding (n = 8). Serum samples were analyzed on an ACE Alera (Alfa Wasswerman) for multiple analytes according to the manufacturer’s instructions.

### Lipid extraction from mouse tissues

Hundred miligram of the liver was homogenized using a mechanical tissue homogenizer. Tissue homogenate was extracted with 18 volumes of hexane:2-propanol (3:2). Samples were vortexed and subsequently centrifuged at 5,000 × g. The supernatant was transferred to a glass conical tube and subsequently washed with one-fifth volume of 0.9% NaCl. Samples were centrifuged at 5,000 × g and the aqueous phase was removed. The lower organic phase was dried down under nitrogen and resuspended in isopropyl alcohol until use.

### Protein extraction

Tissues were dissected and cleaned of adhering fat and soft tissues. They were then washed in ice-cold PBS to remove blood from tissues, snap-frozen in liquid nitrogen, and stored at −80°C until further processing.

Total cell lysates were prepared by homogenization of tissues in radioimmune precipitation buffer containing phosphatase and protease inhibitors followed by the removal of tissue debris by centrifugation. Protein concentrations were determined using the Pierce™ BCA Protein Assay Kit.

### Western blotting

Cell lysates were resuspended in protein sample buffer and incubated at 95°C for 10 min. Protein levels in cell lysates were determined using the Pierce BCA protein assay kit (ThermoFischer Scientific). The protein levels of GAPDH and protein of interest within each panel were obtained from the same nitrocellulose blot by stripping of the blot. Each panel represents a separate Western blot from an individual SDS-PAGE gel. We performed densitometry using ImageJ, which is recommended by the *J. Lipid Res*., on the entire cohort to give the most accurate and statistically relevant protein level ± STD.

Twenty five microgram of protein was loaded in each well and proteins were resolved using a 4%–20% SDS-PAGE gel and transferred to nitrocellulose. Membranes were blocked for 1 h overnight with TBST containing 10% milk and subsequently washed five times with TBST (Tris-buffered saline, 0.1% Tween 20). Washed membranes were incubated with primary antibodies overnight at room temperature. Membranes were washed for 10 min, five times, and with TBST another five times, and subsequently incubated with secondary antibodies for 1–4 h. After five washes with TBST for 10 min each, membranes were incubated in the chemiluminescent agent (Cytiva, Amersham ECL Prime Western Blotting Detection Reagent) and exposed for 2–5 min. β-actin (Abcam) or GAPDH were used as loading controls. Western blots shown in the results section are from three mice and are representative of data obtained from eight mice. The quantitative relative densitometry units were calculated from all 8 mice.

### Hydroxyproline assay

Hydroxyproline levels were determined as previously described (31). Liver tissues were digested in autoclave-safe screw-top tubes in 50 mM K_3_PO_4_ (pH 7) containing 5 mM EDTA, 5 mM N-acetyl-L-cysteine in a final volume of 100 ml. 100 ml of 4N NaOH was added, and samples were autoclaved at 120°C and 15psi for 15 min. Samples were cooled to room temperature and brought to pH 7 using 4N HCL. 625 ml of Chloramine T solution (0.05 Chloramine-T in 74% v/v H2O/26% v/v 2-propanol/0.629 M NaOH/0.140 M citric acetate (anhydrous/0.112 M acetic acid (glacial) was next added. Samples were left at room temperature for 20 min and then 625 ml of Ehrlich’s solution (1M DMAB in 30% v/v HCL/70%v/v 2-propanol) was added. Samples were vortexed immediately and incubated at 65°C in a water bath for 20 min. The reaction was stopped by immersing tubes in ice water. Hydroxyproline levels were determined in 96-well plates by reading absorbance values between 550 and 565 nm.

### Cell-based bioassay for TGF-β1 activity

Total and active TGFb were determined using a cell-based assay as previously described using a commercial assay kit (BPS Bioscience, Cat# 60544).

### Histology and computer-aided image processing

The left lobes of multiple livers (n = 8) that were used for histology staining were stored in 10% neutral buffered saline. Tissues were embedded in parafilm and sectioned at 5 mm. Sections were mounted onto slides, dehydrated by several rounds of ethanol dehydration, and delipidated. Liver sections were stained with hematoxylin (Leica Biosystems) and eosin (Leica Biosystems) or picrosirius red (Polyscience Inc.).

Computer-aided digital scanning was performed using a proprietary machine-learning algorithm (Reveal Sciences). A pdf file is included in the supplemental methods describing how the algorithm analyses are used to calculate values for the levels of micro- and macrovesicular steatosis, macrophage infiltration, hepatocyte ballooning, presence of MDBs, and level of fibrosis (*supplemental pdf, Reveal Biosciences Histology*).

### Apoptosis protein microarray analysis

The levels of phosphorylated or cleaved apoptotic factors were determined using the Mouse Apoptosis Signaling Pathway Array C1 from RayBiotech (Peachtree Corners, GA) using the manufacturers' protocol. The data points represent three independent arrays of the same tissue samples performed in duplicate (n = 3).

### Cytokine protein microarray analysis

The levels of 114 cytokines were determined using the Proteome Profiler Mouse XL Cytokine Array from R & D Systems using the manufacturers protocol. The data points represent three independent arrays of the same tissue samples performed in duplicate (n = 3).

### RNA isolation

Total RNA was isolated from liver tissue with the RNeasy® Mini kit (Qiagen) following the manufacturer’s instructions. Briefly, a Bullet Blender® 24 Gold (Next Advance) was used to homogenize murine liver tissue in RLT lysis buffer. 200-proof (100%) ethyl alcohol (Pharmco by Greenfield Global) was then added, and the samples were applied to a RNeasy column. RNA was then washed with RW1 buffer, followed by an on-column DNase digestion with a RNase-Free DNase Set (Qiagen). RNA was then washed with RNeasy RW1 and RPE buffers. Total RNA was recovered in RNase-free water, quantified by a NanoDrop® ND-1000 spectrophotometer (ThermoFisher Scientific, Waltham, MA), and stored at −80°C.

### *q*RT-PCR analysis

Total RNA was extracted from liver tissue using the RNeasy Plus Universal kit (Qiagen, Germantown, MD) and subjected to DNAse treatment using the RNase-free DNase kit (Qiagen, Germantown, MD). RNA was reversed transcribed using the QuantiTect Reverse Transcription kit (Qiagen, Germantown, MD) and subjected to PCR using the Power SYBR™ RNA-to-C_T_™ 1-step kit (ThermoFisher Scientific (Waltham, MA.). Results were expressed as log_2_fold change of mice fed the CDAA-HFD versus mice fed the chow diet.

### RNASeq analysis

Hepatic transcriptome analysis was performed by RNA sequencing of RNA extracts from 15 mg of liver tissue. RNA quantity was measured using Qubit® (Thermo Scientific). Total RNA quality was determined using a 4150 TapeStation System. An RNA integrity (RIN) score > 7 (10 is maximal) was required for sequencing. RNA libraries were prepared using Illumina Stranded Total RNA Prep and the Ribo-Zero™ Plus” kit (Illumina). Libraries were sequenced on the NextSeq 550 (Illumina) with NextSeq 500/550 High Output Kit v2.5 (2 × 75 Cycles) (Illumina). Reads were aligned to the Ensembl Mus mm39 musculus genome using STAR 2.7 (32). Differential gene expression analysis was performed with DEseq2 (v 1.26.0) (33). Genes with a Benjamini and Hochberg adjusted *P* ≤ 0.05 (5% False Discovery Rate) were regarded as statistically significantly regulated. Enrichment analysis of Gene Ontology pathways was performed using the cluster Profiler package for R (34). The WikiPathway 2023 and Reactome 2022 were retrieved and used for gene annotation enrichment analysis.

### Bile acid extraction

Samples were homogenized by adding one steel bead to 20 mg of solid liver samples, followed by adding 10 μl of 1 μg/ml internal standard working solution and 200 μl of 20% methanol in acetonitrile. After samples were homogenized, they were centrifuged at 2500 rpm for 10 min, and placed at −20°C for 10 min. They were then centrifuged at 4°C at 12,000 rpm for 10 min and supernatants were collected and dried down in a concentrator. Dried samples were reconstituted in 100 μl of 50% methanol-water solution, and were analyzed by LC-MS/MS.

### Bile acid analysis

Samples were analyzed using an LC-ESI-MS/MS system (UPLC, ExionLC™ A, https://sciex.com/; MS, QTRAP® 6500+, https://sciex.com/). The analytical conditions were as follows, HPLC: column, Waters ACQUITY UPLC HSS T3 C18 (100 mm × 2.1 mm i.d., 1.8 μm); solvent system, water with 0.01% acetic acid and 5 mmol/L ammonium acetate (A), acetonitrile with 0.01% acetic acid (B); The gradient was optimized at A/B 95:5 (V/V) at 0 min, A/B 60:40 (V/V) at 0.5 min, 50:50 (V/V) at 4.5 min, 25:75 (V/V) at 7.5 min, 5:95 (V/V) at 10 min, 95:5 (V/V) at 12.0 min; flow rate, 0.35 ml/min; temperature, 40°C; injection volume: 3 μl. The effluent was alternatively connected to an ESI-triple quadrupole-linear ion trap (QTRAP)-MS.

Linear ion trap (LIT) and triple quadrupole (QQQ) scans were acquired on a triple quadrupole-linear ion trap mass spectrometer (QTRAP), QTRAP® 6500+ LC-MS/MS System, equipped with an ESI Turbo Ion-Spray interface, operating in negative ion mode and controlled by Analyst 1.6.3 software (Sciex). The ESI source operation parameters were as follows: ion source, ESI-; source temperature 550°C; ion spray voltage (IS) −4500 V; curtain gas (CUR) was set at 35 psi, respectively. Bile acids were analyzed using scheduled multiple reaction monitoring (MRM). Data acquisitions were performed using Analyst 1.6.3 software (Sciex). Multiquant 3.0.3 software (Sciex) was used to quantify all metabolites. Mass spectrometer parameters including the declustering potentials (DP) and collision energies (CE) for individual MRM transitions were done with further DP and CE optimization. A specific set of MRM transitions was monitored for each period according to the metabolites eluted within this period.

### Statistics

*In vivo* data were analyzed using a two-way ANOVA analysis (mixed model) with Dunnett’s post hoc compared to chow-fed mice (n = 8). Data are presented as an average with a standard error of the mean. Densitometry statistical data were obtained using a paired *t* test and presented as an average with a standard deviation of the mean (n = 8). Microarray data is presented as an average with the standard deviation of the mean using a paired *t* test (n = 4). Bile acid indices values are mean ± STD.

## Results

### *HuMgat2* mice fed a CDAA-HFD have MASLD that responds to elafibranor

Male *mMgat2* and *HuMgat2* mice were fed a chow diet or a CDAA-HFD for a total of 24 weeks. Eight weeks into the study, one group fed the CDAA-HFD diet was switched to a CDAA-HFD containing 30 mg/kg elafibranor (CDAA-HFD + ELA) until the end of the study. The two remaining cohorts were continued on the CDAA-HFD until the end of the study. Elafibranor was formulated into the chow.

**Fig. 1:**
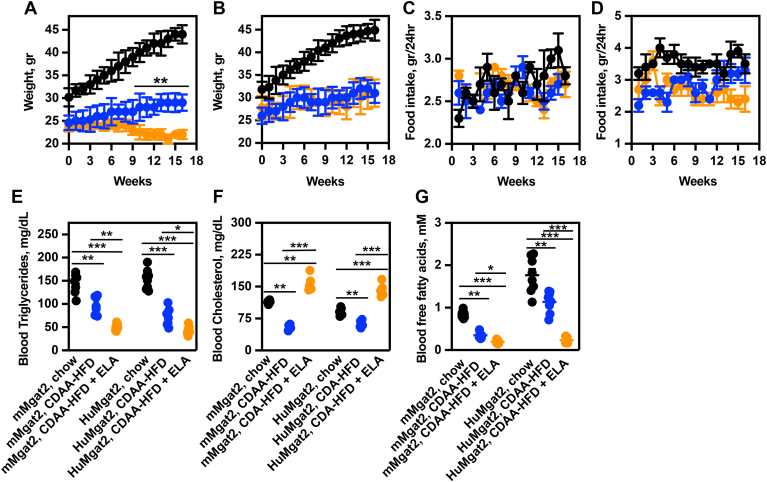
*HuMgat2* mice develop mixed hyperlipidemia when fed a CDAA-HFD. Male *mMgat2* and *HuMgat2* mice were fed a chow diet for the entire study (black circles), a CDAA-HFD for the entire study (blue circles), or a CDAA-HFD for 8 weeks then switched to a CDAA-HFD plus elafibranor (ELA) diet for 16 weeks (orange circles) (n = 8). The study was terminated at 24 weeks (*t = 0, start of elafibranor treatment*). (A) body weight of *mMgat2* mice over time, (B), body weight of *HuMgat2* mice over time, (C), food intake of *mMgat2* mice over time, (D), food intake of *HuMgat2* mice over time, (E) blood triglycerides levels of *mMgat2* and *HuMgat2* mice, (F) blood cholesterol levels of *mMgat2* and *HuMgat2* mice, (G) blood free fatty acids levels of *mMgat2* and *HuMgat2* mice. Two way ANOVA (mixed model) with Dunnett’s post hoc compared to chow-fed mice was used for statistical analysis. Data are mean ± S.T.D ∗*P* ≤ 0.01; ∗∗*P* ≤ 0.001; ∗∗∗*P* ≤ 0.0001.

*mMgat2* and *HuMgat2* mice fed chow gained weight at approximately the same rate (Fig. 1A and B, black circles), whereas no weight gain was observed on the CDAA-HFD (Fig. 1A, blue circles). *mMgat2* mice fed the CDAA-HFD containing elafibranor began to lose weight at 9 weeks (1 week post-treatment initiation) that leveled off at 13 weeks (Fig. 1A, blue circles vs. orange circles). *HuMgat2* mice fed the same diet did not lose weight (Fig. 1B, blue circles vs. orange circles). Weekly food intake was similar for *mMgat2* and *HuMgat2* mice regardless of diet (Fig. 1C and D).

*mMgat2* and *HuMgat2* mice fed the CDAA-HFD had decreased levels of blood triglycerides, cholesterol, and free fatty acids compared to mice fed chow (Fig. 1E–G, blue circles vs. black circles). Treating mice with elafibranor further reduced triglycerides and free fatty acids levels to less than those seen in mice fed chow or CDAA-HFD (Fig. 1E and G, orange circles vs. blue and black circles), but increased blood cholesterol levels by ∼2-fold relative to mice fed chow (Fig. 1F, blue circles vs. orange circles), and ∼3-fold relative to mice fed the CDAA-HFD (1F, orange circles vs. blue circles). It is worth noting that the free fatty acids levels in the blood of *HuMgat2* mice fed chow were ∼1.8-fold higher than those seen for *mMgat2* mice fed the same diet (Fig. 1G, black circles).

**Fig. 2:**
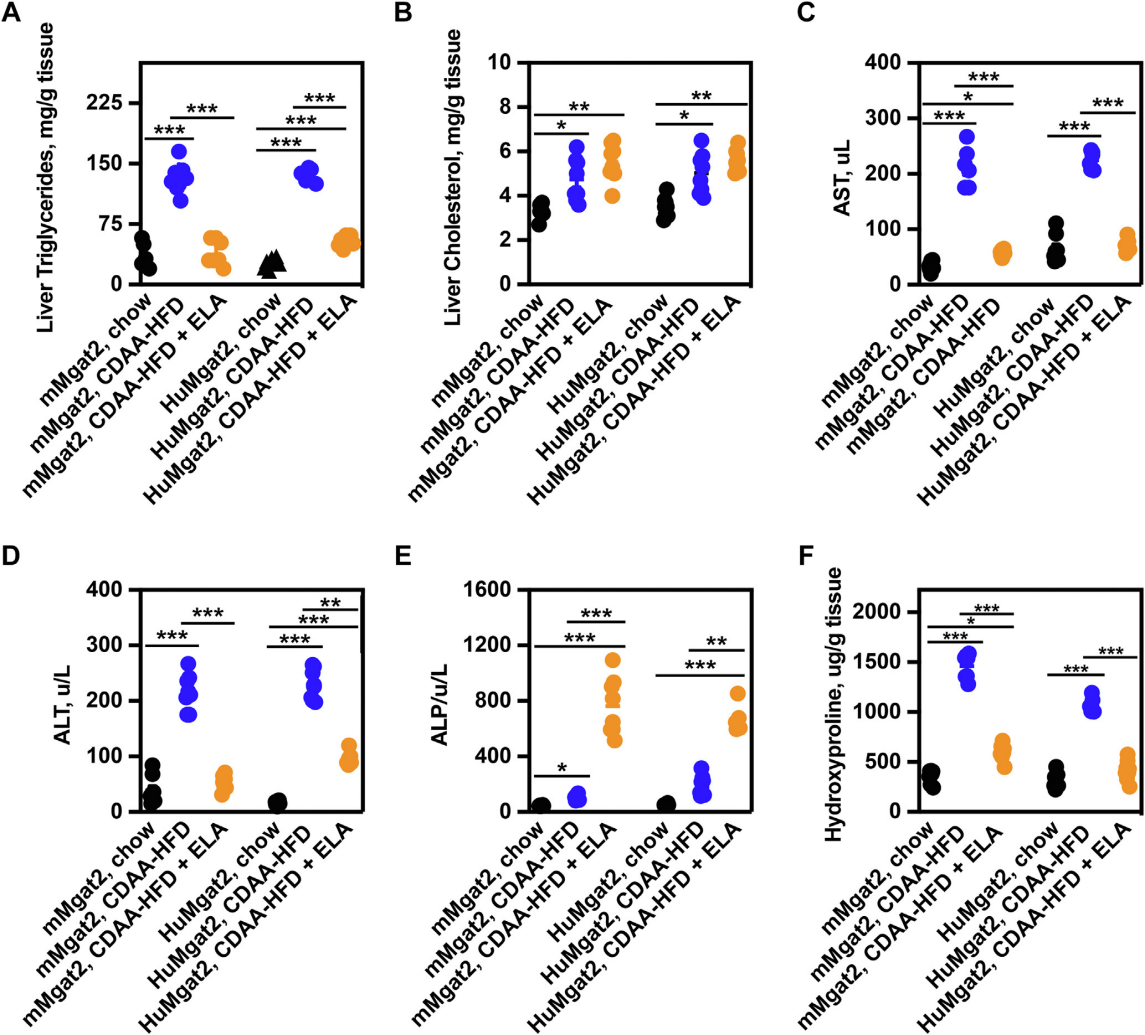
*HuMgat2* mice develop hepatic steatosis and liver damage on a CDAA-HFD. Mice were treated as described in figure legend 1. Livers from *mMgat2* and *HuMgat2* mice were used for analysis (n = 8). (A), liver triglycerides levels of *mMgat2* and *HuMgat2* mice, (B) liver cholesterol levels of *mMgat2* and *HuMgat2* mice, (C) Plasma AST levels of *mMgat2* and *HuMgat2* mice, (D) Plasma ALT levels of *mMgat2* and *HuMgat2* mice, (E) Plasma ALP levels of *mMgat2* and *HuMgat2* mice, (F), hydroxyproline levels of *mMgat2* and *HuMgat2* mice. Two way ANOVA (mixed model) with Dunnett’s post hoc compared to chow-fed mice was used for statistical analysis (n = 8). Data are mean ± S.T.D ∗*P* ≤ 0.01; ∗∗*P* ≤ 0.001; ∗∗∗*P* ≤ 0.0001.

*mMgat2* and *HuMgat2* mice fed the CDAA-HFD had elevated liver triglycerides and cholesterol levels (Fig. 2A and B, blue circles vs. black circles). Treating mice with elafibranor reduced triglycerides levels (Fig. 2A, blue circles vs. orange circles) but cholesterol levels remained elevated (Fig. 2B, blue circles vs. orange circles).

### *HuMgat2* mice show signs of lipotoxic-dependent liver dysfunction

The accumulation of triglycerides in the liver causes lipotoxicity, which is a major risk factor for developing MASLD/MASH (3, 4). The relationship between elevated AST, ALT, and ALP liver enzyme levels and increased liver dysfunction is well established (35). Thus, we determined the levels of these enzymes in mice fed the various diets.

All three enzyme levels were elevated in *mMgat2* mice fed the CDAA-HFD (Fig. 2D–F, blue circles vs. black circles). *HuMgat2* mice fed the CDAA-HFD diet had elevated levels of AST and ALT (Fig. 2D and F, blue circles vs. black circles). AST and ALT levels were reduced when both cohorts were treated with elafibranor (Fig. 1D and F, orange circles vs. blue circles), whereas elafibranor treatment caused increases in ALP levels compared to mice fed chow (Fig. 2E, orange circles vs. black circles).

We next measured the levels of hydroxyproline, a major constituent of collagen that accumulates in livers of mice with MASH (36). Livers from *mMgat2* and *HuMgat2* mice fed the CDAA-HFD had elevated levels of hydroxyproline over mice fed chow (Fig. 2F, blue circles vs. black circles), which elafibranor treatment reduced (Fig. 2F, blue circles vs. orange circles).

### Livers of *HuMgat2* mice show histological signs of MASH with macrophage infiltration and fibrosis

**Fig. 3:**
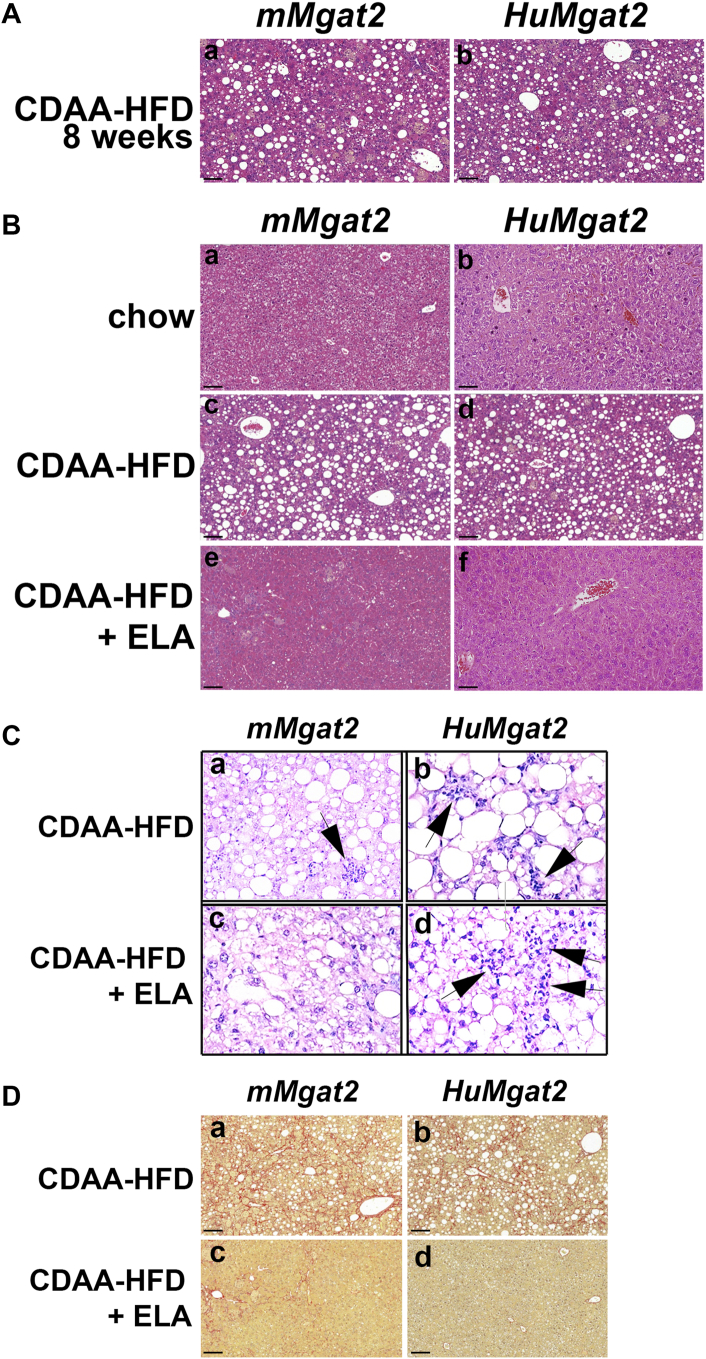
*HuMgat2* mice develop MASH with fibrosis on a CDAA-HFD. Liver sections from *Mgat2* and *HuMgat2* mice were stained with hematoxylin and eosin (A–C) or picrosirius red (D) for visualizing steatosis, macrophage inflammation, and fibrosis by microscopy. (A*a & b*), H&E staining of liver sections from *mMgat2* and *HuMgat2* mice that had been fed a CDAA-HFD for 8 weeks in the absence of elafibranor treatment. (B*a & b*), H&E stained liver sections from *mMgat2* and *HuMgat2* that had been fed a chow diet for 24 weeks, (B*c & d*), H&E stained liver sections from *mMgat2* and *HuMgat2* that had been fed a CDAA-HFD diet for 24 weeks, (B*e & f*), H&E stained liver sections from *mMgat2* and *HuMgat2* that had been fed a CDAA-HFD diet for 8 weeks and subsequently treated with 30 mg/kg elafibranor for 16 weeks, (*Ca &* b), H & E stained liver sections from *mMgat2* and *HuMgat2* that had been fed a CDAA-HFD diet for 24 weeks, (C*c & d*), H&E stained liver sections from *mMgat2* and *HuMgat2* that had been fed a CDAA-HFD diet for 8 weeks and subsequently treated with 30 mg/kg elafibranor for 16 weeks. Panels C*a*-C*d* have been brightened and sharpened using Adobe photoshop 2022 to enhance immune cell visualization. (D*a & b*), Picrosirius red stained liver sections from *mMgat2* and *HuMgat2* that had been fed a CDAA-HFD diet for 24 weeks, (D*c & d*), Picrosirius red stained liver sections from *mMgat2* and *HuMgat2* that had been fed a CDAA-HFD diet for 8 weeks and subsequently treated with 30 mg/kg elafibranor for 16 weeks. Bar = 100 μm. panels are representative images of 5 independent mice.

We used histological staining and microscopy to examine the extent of liver tissue damage. Eight weeks into the study and at the initial start of elafibranor treatment, the livers of *mMgat2* and *HuMgat2* mice fed the CDAA-HFD had developed MASLD but lacked any signs of MASH (Fig. 3A, *a* & *b*). At the end of the study, hepatocytes of livers from mice fed chow had a normal cell morphology that showed no signs of lipid deposition, hepatic steatosis, macrophage infiltration, or fibrosis (Fig. 3B, *a* & *b*). Hepatocytes from mice fed the CDAA-HFD had a high level of intracellular fat accumulation with associated micro- and macrovesicular steatosis (Fig. 3B, *c* & d) that elafibranor cleared (Fig. 3C, *e* & *f*). We observed macrophage infiltration in livers of *mMgat2* and *HuMgat2* mice fed the CDAA-HFD (Fig. 3C, *a* vs. *b*; arrows). *mMgat2* mice treated with elafibranor showed reduced signs of infiltration (Fig. 3C, *c*), whereas *HuMgat2* mice showed only a slight reduction (Fig. 3C, *d*). Fibrosis was also visualized in both cohorts fed the CDAA-HFD (Fig. 3D, *a* & *b*) that was significantly reduced by treating with elafibranor (Fig. 2D, *c* & *d*). Using staging values for steatosis, macrophage infiltration, and fibrosis, a NAS of 6 was calculated for both cohorts indicating the presence of MASH.

We used computer-aided histological to quantitate the amount of liver damage (37, 38, 39) (Reveal Biosciences, San Diego, CA). Similar areas of tissue were used for calculations (n = 5).

**Fig. 4:**
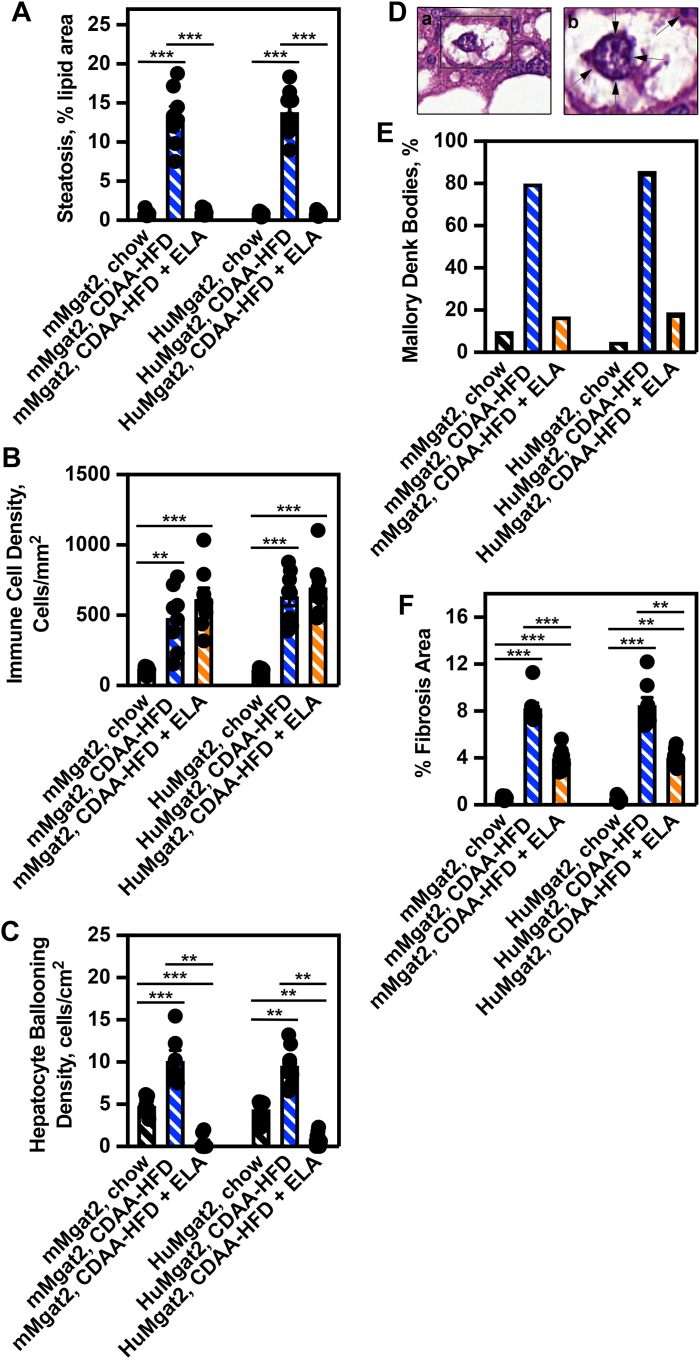
AI-aided histology reveals the presence of hepatocyte cell ballooning and elevated Mallory-Denk aggregate body levels in *HuMgat2* mice with MASH. AI-aided quantitative histological analysis was performed on stained liver sections using Reveal Bioscience algorithm software (n = 5). Data was obtained from, and calculations were based on examination of the same surface area of each individual slide. (A), percentage steatosis (lipid deposition) per surface area, (B), immune cell density per surface area, (C), level of hepatocyte ballooning density per surface area, (D) histological liver sections that were enlarged to visualize Mallory-Denk bodies (arrows), (E), percentage of cells having 1–5 Mallory-Denk bodies, (F), percentage fibrosis per surface area. Two way ANOVA (mixed model) with Dunnett’s post hoc compared to chow-fed mice was used for statistical analysis. Data are mean ± S.T.D. ∗∗*P* ≤ 0.001; ∗∗∗*P* ≤ 0.0001.

Very little hepatocyte fat deposition was quantified for *mMgat2* and *HuMgat2* mice fed the chow diet (∼1%) (Fig. 4A, black hatched bars), with cells showing minimal signs of micro- and macrovesicular steatosis. Livers from mice fed the CDAA-HFD had a fat percentage of ∼15% (Fig. 4A, blue hatched bars) with a high level of macrovesicular steatosis (>80%). Elafibranor treatment decreased fat deposition by >95% (Fig. 4A, orange hatched bars) and macrovesicular steatosis (>85%). Livers from mice fed the CDAA-HFD showed increased hepatocyte immune cell density (Fig. 4B, blue hatched bars) and increased immune cell numbers compared to mice fed chow (>50-fold). Here, elafibranor treatment was ineffective in reducing infiltration (Fig. 4B, orange hatched bars vs. blue hatched bars).

We determined the level of hepatocyte ballooning and the presence of Mallory-Denk bodies using AI histology (Fig. 4C and D). *mMgat2* and *HuMgat2* mice fed the CDAA-HFD had a 2-fold increase in the number of lipid-ladened ballooning hepatocytes over numbers calculated for mice fed the chow diet (Fig. 4C, blue hatched bars), with elafibranor treatment significantly reducing these numbers (Fig. 4C, orange hatched bars).

Very few MDBs (Fig. 4D and E) were observed in hepatocytes from mice fed chow, with ∼10% of cells having 1–5 bodies per area analyzed (Fig. 4E, chow). This number rose to ∼80% in mice fed the CDAA-HFD (Fig. 4E, blue hatched bars). The number of cells with MDBs was reduced by elafibranor treatment (Fig. 4E, blue hatched bars vs. orange hatched bars).

There were clear signs of fibrosis in the livers of mice fed the CDAA-HFD (>8%) that were not present in the livers of those fed chow (Fig. 4F, blue hatched bars vs. black hatched bars). Elafibranor reduced these levels by ∼40% (Fig. 4F, blue-hatched bars vs. orange-hatched bars).

### Livers of *HuMgat2* mice with MASH show defects in autophagic clearance of pp62-containing protein aggregates

Phosphorylated p62/sequestosome-1 (pp62) is crucial for forming and stabilizing MDBs (40). It acts as a nucleating factor for the formation of ubiquitinated protein aggregates that make up the core of MDBs (40). Multiple cell lines accumulate pp62 aggregates during lipotoxic conditions (41, 42) and high levels are found in the livers of mice and humans with MASH (43, 44, 45).

TBK1-dependent p62^Ser403^ phosphorylation stimulates pp62 degradation and its associated ubiquitinated cargo under normal autophagic conditions (46). Therefore, very little pp62 should be present in cells during normal proliferation.

**Fig. 5:**
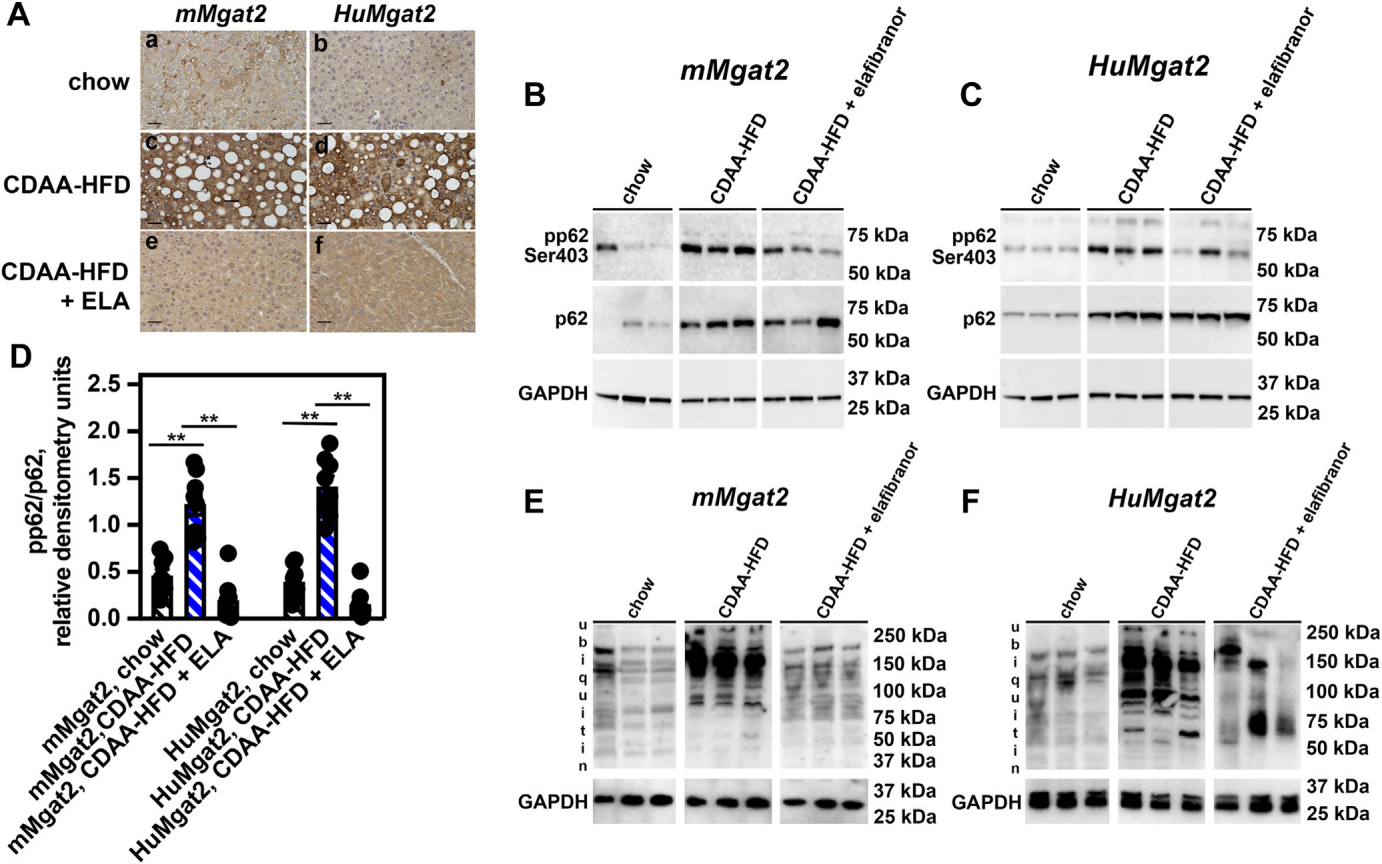
*HuMgat2* mice with MASH accumulate phosphorylated p62/sequestosome-1 ubiquitinated protein aggregates. (A) liver sections from *mMgat2* and *HuMgat2* mice fed the various diets were stained with anti-p62 antibodies and visualized by IHC (n = 5). (*a & b*), mice fed a chow diet, (*c & d*), mice fed a CDAA-HFD, (*e & f*), mice fed a CDAA-HFD + 30 mg/kg elafibranor (ELA). (B and C), western analysis of pp62^Ser403^ and total p62 protein levels in livers from *mMgat2* and *HuMgat2* mice fed the various diets. (D), quantitative densitometry of protein levels of pp62^Ser403^ and total p62 in livers from *mMgat2* and *HuMgat2* mice fed the various diets (n = 8). (E and F), western analysis of the levels of ubiquitin protein aggregates. GAPDH was used as a loading control. IHC panels are representative of images obtained from 5 individual mice. Densitometry statistical data were obtained using a paired *t* test. Data are mean ± S.T.D. ∗∗*P* ≤ 0.001.

We observed very little pp62 levels in the livers of *mMgat2* and *HuMgat2* mice fed chow using IHC staining (Fig. 5A, *a & b*). Levels became markedly increased in mice fed the CDAA-HFD (Fig. 5A, *a & b* vs. *c & d*) that elafibranor treatment efficiently decreased (Fig. 5A, *c & d* vs. *e & f*).

Low levels of p62 and pp62 were detected in livers from mice fed chow using western blotting (Fig. 5B–D, black hatched bars). Levels became elevated when mice were fed the CDAA-HFD (Fig. 5B–D, blue hatched bars). While elafibranor did not reduce total p62 levels, it did decrease pp62 levels to like those detected in mice fed chow (Fig. 5B–D, orange hatched bars vs. blue hatched bars).

Sustained pp62 levels should lead to the accumulation of poly-ubiquitinated proteins. We detected low levels of ubiquitinated proteins with varying molecular weights in the livers of mice fed chow (50 to 250 kDa) (Fig. 5E and F, chow). These accumulated when mice were fed the CDAA-HFD (Fig. 5E and F, CDAA-HFD vs. chow). Treating mice with elafibranor resulted in a marked reduction in these species (Fig. 5E and F, CDAA-HFD + elafibranor vs. CDAA-HFD).

Since autophagic-driven degradation removes pp62-associated protein aggregates we hypothesized their accumulation in the livers of mice fed the CDAA-HFD may result from defects in autophagy. During autophagy LC3-I becomes lipidated, which generates LC3-II (47). LC3-II plays roles in the formation of the autophagosome and degradation of ubiquitinated protein cargo within autophagolysosomes (48). A high LC3-II:LC3-I protein ratio correlates with active autophagic flux (44). We used western blotting and determined LC3-I and LC3-II protein levels to calculate LC3-II:LC3-I ratios as a measure of the level of autophagic flux in the livers of mice fed the various diets.

**Fig. 6:**
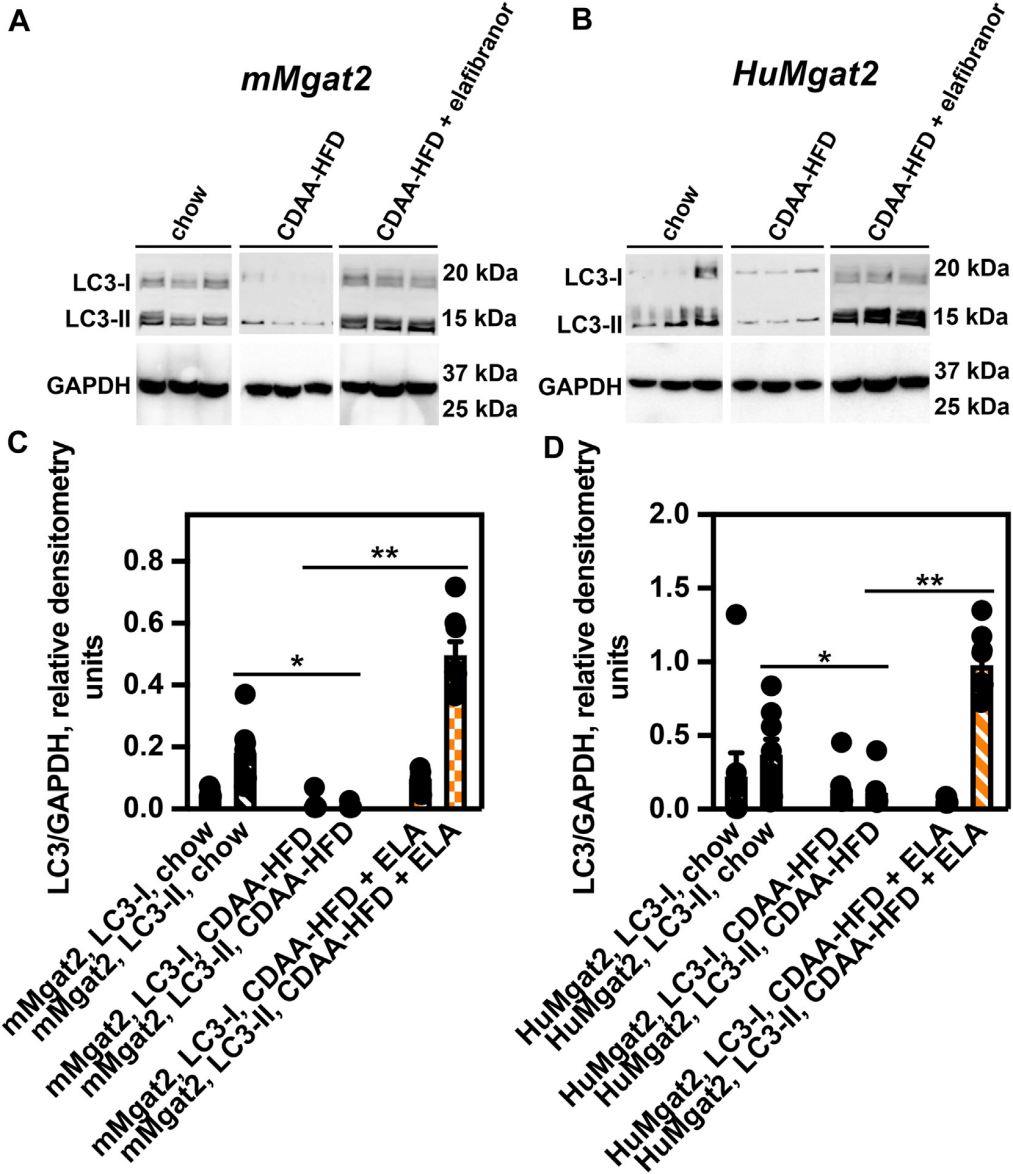
Livers of HuMgat2 mice with MASH have defects in autophagy that may contribute to Mallory-Denk bodies formation. The levels of the autophagy marker proteins, LC3-I and LC3-II were determined by western analysis. (A), liver LC3-I and LC3-II levels from *mMgat2* mice fed the various diets. (B), liver LC3-I and LC3-II levels from *HuMgat2* mice fed the various diets. (C) quantitative densitometry of protein levels of LC3-I and LC3-II in livers from *mMgat2* mice fed the various diets (n = 8). (D) quantitative densitometry of protein levels of LC3-I and LC3-II in livers from *HuMgat2* mice fed the various diets (n = 8). GAPDH was used as a loading control. Densitometry statistical data were obtained using a paired *t* test. Data are mean ± S.T.D. ∗*P* ≤ 0.01; ∗∗*P* ≤ 0.001.

Livers from *mMgat2* mice fed chow had increased LC3-II to LC3-I levels and a calculated LC3-II:LC3-I ratio of ∼3.7 using quantitative densitometry analyses (Fig. 6A and C). This ratio dropped to ∼0.75 in the livers of mice fed the CDAA-HFD, where we saw very little LC3-I and LC3-II protein levels, suggesting defects in autophagy (Fig. 6A and C). A robust increase in LC3-II levels were detected in the livers from mice treated with elafibranor (LC3-II:LC3-I ratio of ∼5.0), strongly suggesting the restoration of normal autophagy (Fig. 6A and C).

The same LC3-II:LC3-I ratio trends were seen for livers from *HuMgat2* mice fed the various diets. Chow-fed mice had a LC3-II:LC3-I ratio of ∼6.0, higher than the ratio of mMgat2 mice due to increased levels of LC3-II (Fig. 6B and D). This ratio fell to ∼ 0.85 when mice were fed the CDAA-HFD (Fig. 6B and D)). Treating with elafibranor increased LC3-II levels resulting in an LC3-II:LC3-I ratio of ∼18, once again suggesting normal autophagic flux.

### TGF-β1 activates hepatic stellate cells in the livers of *HuMgat2* mice with MASH

HSCs are a small population of quiescent cells in the liver (49). They become activated during liver injury and transition into myofibroblast-like cells that are critical for initiating fibrosis through their expression and secretion of type I and type III collagens driven by TGF-β1-dependent signaling (49, 50). The presence of fibrosis in the livers of *mMgat2* and *HuMgat2* mice fed CDAA-HFD would predict HSCs were in their active state. To test this, we first determined if the initial signaling cytokine for HSC activation, TGF-β1, was present. Total and active TGF-β1 levels were measured using previously developed cell-reporter bioassays (51, 52).

**Fig. 7:**
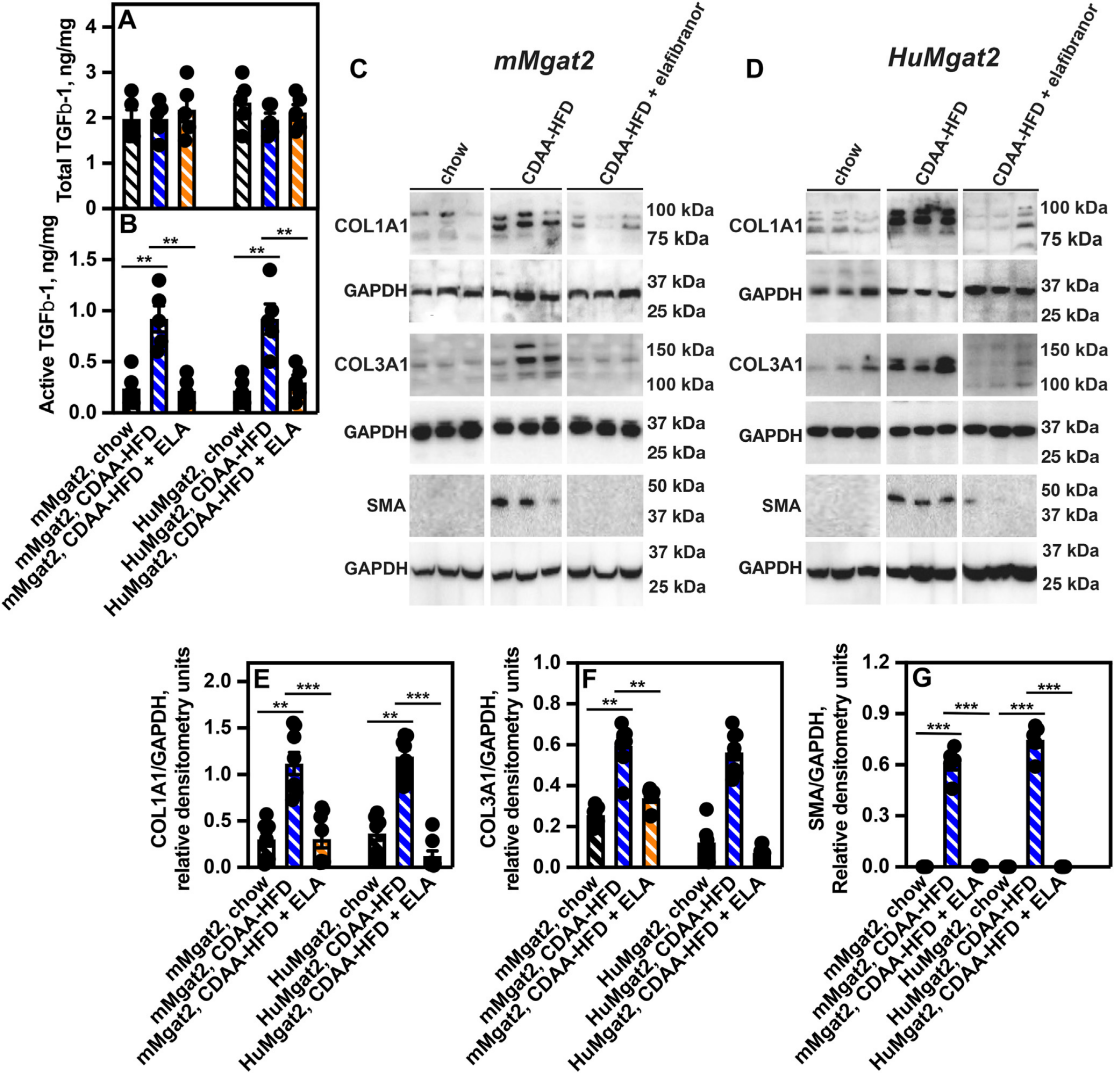
TGF-1β drives hepatic stellate cell activation resulting in the increase of collagen deposition in the livers of *HuMgat2* mice with MASH. The levels of total and active were determined using established cell-based assays. Collagen protein levels were determined by western analysis. (A), the levels of total TGF-1β, (B), the levels of active TGF-1β, (C), protein levels of the collagens, COL1A1 and COL1A3, and αSMA in livers from *mMgat2* mice, (D), protein levels of the collagens, COL1A1 and COL1A3, and αSMA in livers from *HuMgat2* mice, (E), quantitative densitometry of protein levels of COL1A1 in livers from *mMgat2* and *HuMgat2* mice fed the various diets (n = 8), (F), quantitative densitometry of protein levels of COL3A1 in livers from *mMgat2* and *HuMgat2* mice fed the various diets (n = 8), (G), quantitative densitometry of protein levels of αSMA in livers from *mMgat2* and *HuMgat2* mice fed the various diets (n = 8). GAPDH was used as a loading control. Densitometry statistical data were obtained using a paired *t* test. Data are mean ± S.T.D. ∗∗*P* ≤ 0.001; ∗∗∗*P* ≤ 0.0001.

Total TGF-β1 constitutes latent and active forms. Total liver TGF-β1 levels did not change in the livers from either cohort under any feeding condition (Fig. 7A), whereas active TGF-β1 levels markedly increased in mice fed the CDAA-HFD (Fig. 7B, blue hatched bars vs. black hatched bars). Elafibranor treatment brought levels down to those seen in mice fed chow (Fig. 7B, orange hatched bars vs. blue hatched bars).

Concomitant increases in COL1A1 and COL3A1 levels were seen in mice fed the CDAA-HFD that were expressing high levels of active TGF-β1 (Fig. 7C–F blue hatched bars vs. black hatched bars). Treating with elafibranor, which reduced active TGF-β1 levels, also reduced COL1A1 and COL3A1 protein levels (Fig. 7C–F, orange hatched bars vs. blue hatched bars). The levels of the myofibroblast marker, αSMA, were also elevated in mice fed the CDAA-HFD (Fig. 7C, D, and G, blue hatched bars vs. black hatched bars) that were reduced by elafibranor (Fig. 7C, D and G, orange hatched bars vs. blue hatched bars).

### Hepatocyte apoptosis drives MASH in *HuMgat2* mice

Increased levels of active cleaved caspase-3 are present in the livers of MASH patients (53). Several studies have reported that *casp3*^*−/−*^ mice are protected from fibrosis (54, 55). As caspase 3 drives hepatocyte apoptosis (56, 57, 58), we determined whether cleaved caspase-3 levels were elevated in the livers of mice fed the CDAA-HFD.

**Fig. 8:**
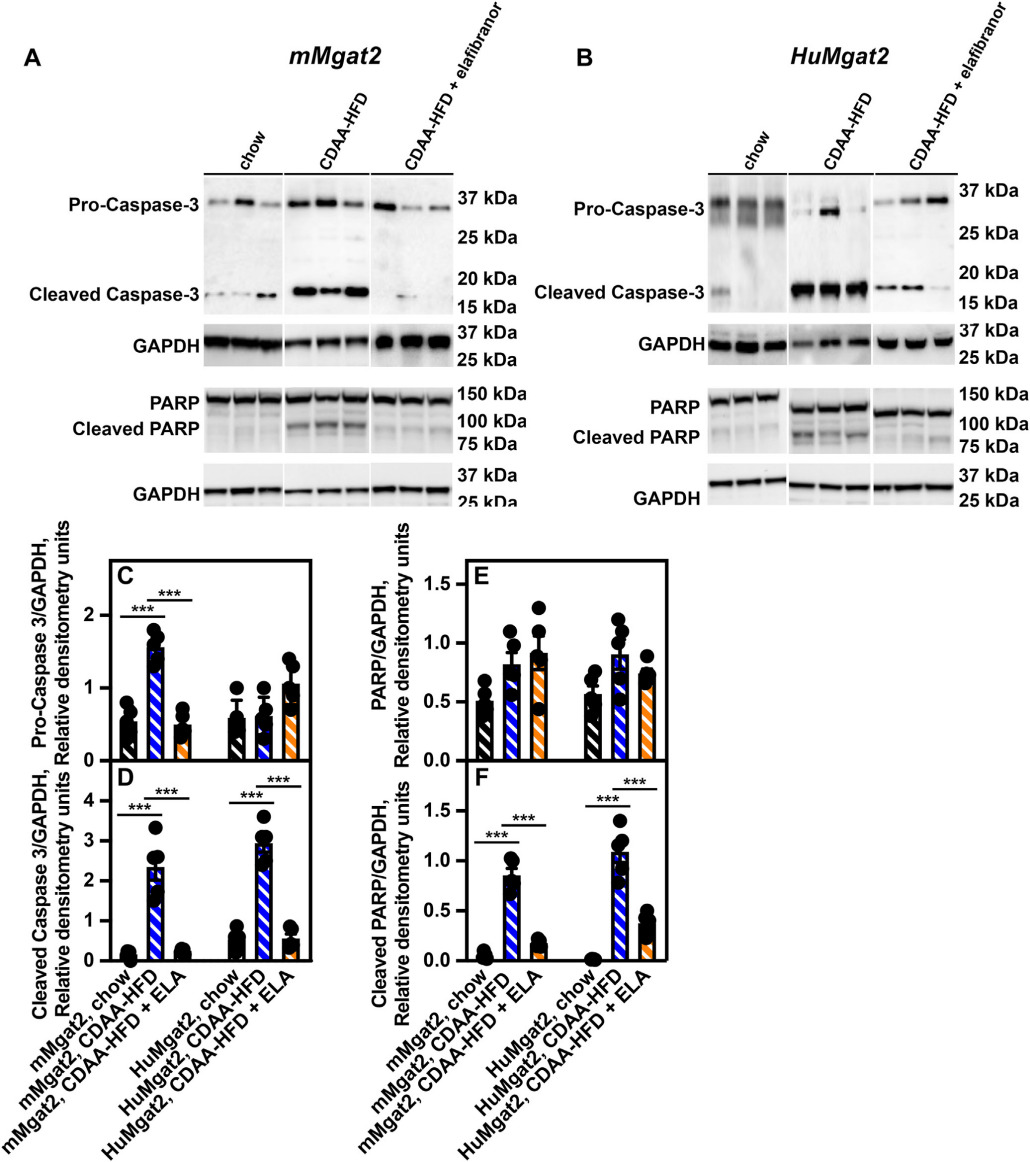
Activated caspase 3 drives apoptosis in liver hepatocytes of *HuMgat2* mice with MASH. cleaved caspase were determined by western analysis. Pro-caspase 3, cleaved caspase-3, PARP1 and cleaved PARP1 were determined by western analysis, (A), protein levels of pro- and cleaved caspase-3, and PARP1 and cleaved PARP1 in livers from *mMgat2* mice, (B), protein levels of pro- and cleaved caspase-3, and PARP1 and cleaved PARP1 in livers from *HuMgat2* mice, (C), quantitative densitometry of protein levels of pro-caspase 3 in livers from *mMgat2* and *HuMgat2* mice fed the various diets (n = 8), (D), quantitative densitometry of protein levels of cleaved caspase 3 in livers from *mMgat2* and *HuMgat2* mice fed the various diets (n = 8), (E), quantitative densitometry of protein levels of PARP1 in livers from *mMgat2* and *HuMgat2* mice fed the various diets (n = 8), (F), quantitative densitometry of protein levels of cleaved PARP1 in livers from *mMgat2* and *HuMgat2* mice fed the various diets (n = 8). GAPDH was used as a loading control. Densitometry statistical data were obtained using a paired *t* test. Data are mean ± S.T.D. ∗∗∗*P* ≤ 0.0001.

Low levels of pro- and cleaved-caspase-3 were detected in the livers from *mMgat2* mice fed chow (Fig. 8A and C, black hatched bars). Both forms markedly increased when mice were fed the CDAA-HFD (Fig. 8A and D, blue hatched bars). Elafibranor treatment decreased pro-caspase-3 levels by 84% in mMgat2 mice (Fig. 8A and D, orange hatched bars).

Normal pro-caspase three levels were detected in the livers of *HuMgat2* mice fed all diets (Fig. 8B and C, black hatched bars). Feeding mice the CDAA-HFD increased cleaved caspase-3 levels by greater than 200-fold (Fig. 8B and C, blue hatched bars), which was reduced by 93% by elafibranor treatment (Fig. 8B and D, orange bars).

PARP1 is a 116 kDa protein that is cleaved during apoptosis by caspase three into 89- and 24-kDa fragments (59, 60). To determine the activation status of caspase-3 protease activity, we quantified the protein levels of the 89-kDa PARP1 fragment in mice fed the various diets.

We did not observe any diet-dependent changes in full-length PARP1 levels in livers from *mMgat2* and *HuMgat2* mice (Fig. 8A, B and E, black hatched bars). Cleaved PARP1 levels were low in mice fed chow, whereas levels in those fed the CDAA-HFD were elevated ∼12- and ∼30-fold in *mMgat2* and *HuMgat2* mice, respectively (Fig. 8A, B and F, black hatched bars vs. blue hatched bars). Treating with elafibranor reduced these cleaved products by 80% and 65% in *mMgat2* and *HuMgat2* mice, respectively (Fig. 8, A–F, orange hatched bars vs. blue hatched bars).

### *HuMgat2* mice with MASH display a unique inflammatory cytokine response when fed the CDAA-HFD

Metainflammation is a term used to describe the chronic macrophage-driven cytokine response seen during MASH (61). Because we observed a high level of macrophage infiltration in the livers of mice fed the CDAA-HFD using AI histology, we profiled the cytokine signatures of livers from *mMgat2* and *HuMgat2* mice fed the various diets using cytokine protein microarrays.

**Fig. 9:**
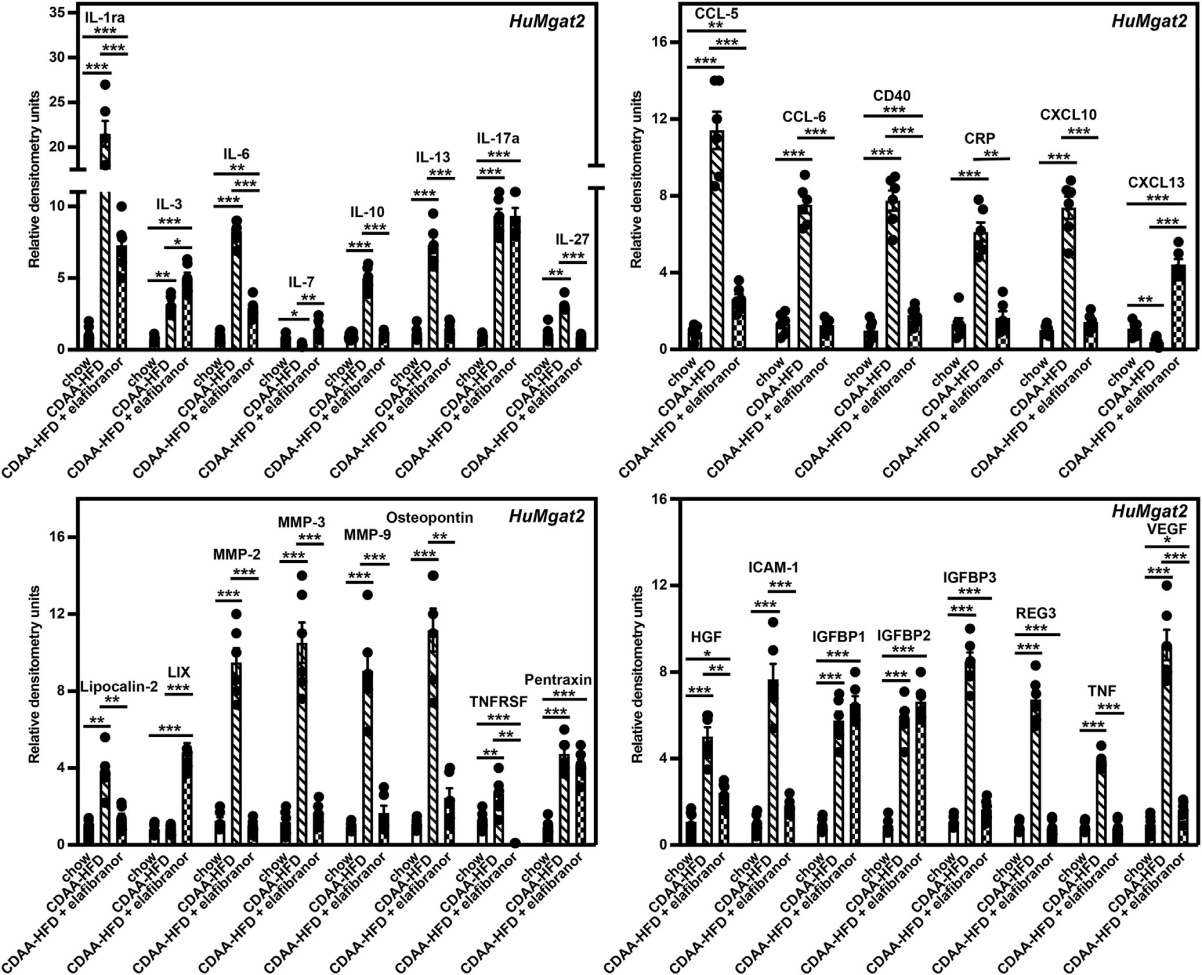
Several key inflammatory markers are elevated in livers from *HuMgat2* mice with MASH. Protein levels of 114 cytokines were determined from three individual livers from *HuMgat2* mice fed the various diets. Levels were determined in duplicate, and all six values were averaged. Proteins levels were compared to protein level control wells on each microarray strip. Densitometry statistical data were obtained using a paired *t* test. Data are mean ± S.T.D. ∗*P* ≤ 0.01; ∗∗*P* ≤ 0.001; ∗∗∗*P* ≤ 0.0001.

**Fig. 10:**
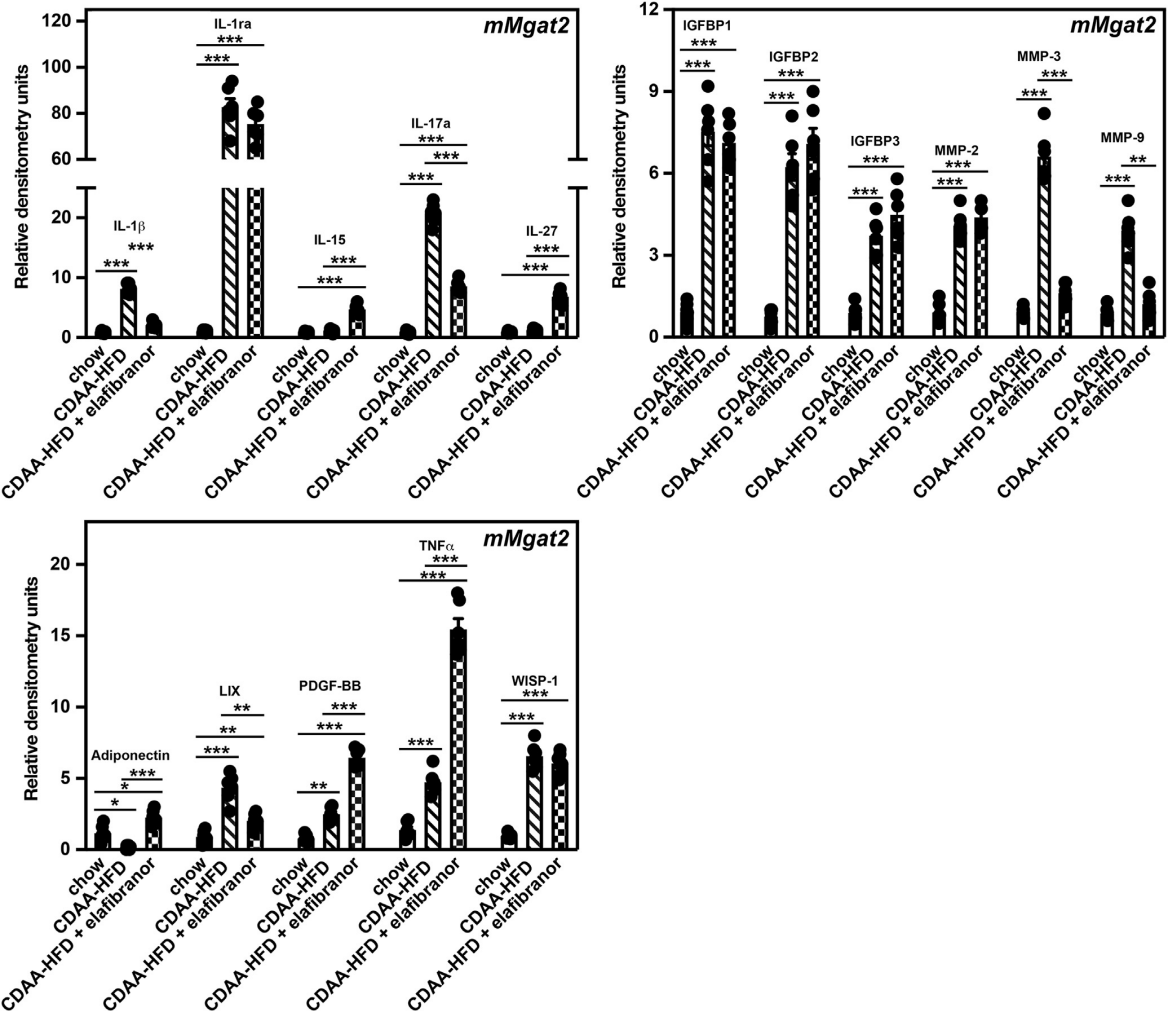
Several key inflammatory markers are elevated in livers from *mMgat2* mice with MASH. Protein levels of 114 cytokines were determined from three individual livers from *mMgat2* mice fed the various diets. Levels were determined in duplicate, and all six values were averaged. Proteins levels were compared to protein level control wells on each microarray strip. Densitometry statistical data were obtained using a paired *t* test. Data are mean ± S.T.D. ∗*P* ≤ 0.01; ∗∗*P* ≤ 0.001; ∗∗∗*P* ≤ 0.0001.

A combined total of 35 of 114 cytokines were detected in the livers of *mMgat2* and *HuMgat2* mice fed a chow diet (Table 1). 19 of 24 cytokines were detected only in *HuMgat2* mice and 5 alone in *mMgat2* mice. CDAA-HFD-fed *mMgat2* and *HuMgat2* mice showed common increases in the levels of 10 cytokines that included the key inflammatory markers, Tnf, IL-1ra and IL-27 (Fig. 9, Fig. 10). Only *HuMgat2* mice responded to elafibranor treatment and reduced the levels of these cytokines. In fact, TNF and IL-27 levels were increased in *mMgat2* mice treated with elafibranor over those seen in mice fed the CDAA-HFD. In the case of IL-27, *mMgat2* mice fed the CDAA-HFD alone had IL-27 levels like chow-fed mice, so the increase was elafibranor specific.

**Table 1:** Cytokine protein expression levels in the livers of *mMgat2* and *HuMgat2* mice fed the chow diet

Protein	*mMgat2*	*HuMgat2*
Adiponectin	*+*^a^	−b
IL-1b	*+*	−
IL-15	*+*	−
PDGF-BB	*+*	−
WISP-1	*+*	−
CCL5	−	+
CCL6	−	+
CD40	−	+
C-Reactive Protein	−	+
CXCL10	−	+
CXCL13	−	+
HGF	−	+
CD54 (ICAM1)	−	+
IL-3	−	+
IL-6	−	+
IL-7	−	+
IL-10	−	+
IL-13	−	+
Lipocalin-2	−	+
Osteopontin	−	+
Osteoprotegerin	−	+
Pentraxin-2	−	+
Reg3G	−	+
VEGF	−	+

Presence or absence of expression was based on the average raw densitometry values relative to negative controls (IMAGEJ software). Three microarray blots were used for a single mouse from each cohort (n = 3), giving 6 data points for determining the presence or absence of expression.

a Detected.

b Absent.

Overall, treating *mMgat2* and *HuMgat2* mice fed the CDAA-HFD with elafibranor was efficacious in decreasing the levels of most cytokines, with a few exceptions. While Cxcl13 levels remained at baseline when *HuMgat2* mice were fed the CDAA-HFD, they became elevated 6-fold upon elafibranor treatment (Fig. 10). The elevated levels of IL-17a (10-fold), IL-3 (5-fold), and pentraxin-2 (5-fold) observed in *HuMgat2* mice fed the CDAA-HFD remained high even with elafibranor treatment. *mMgat2* mice treated with elafibranor showed a sustained increase in the level of adiponectin (3-fold), Pdgf-BB (6-fold), IL-15 (4-fold), and Wisp (7-fold) (Fig. 9). IL-15 levels were not induced by the CDAA-HFD alone but were when mice were treated with elafibranor.

### Elafibranor attenuates MASH transcriptomic gene expression

We performed RNASeq analysis on livers from *mMgat2* and *HuMgat2* mice fed the various diets. Gene expression data was generated by combining the expression values from four individual livers and calculating an average.

Elafibranor treatment efficiently dampened MASH-driven gene expression in livers from *mMgat2* and *HuMgat2* mice fed the CDAA-HFD, as a large subset of the top 100 up and downregulated transcripts returned to near chow-fed levels, as seen by their log_2_-fold changes approaching 1 (supplemental Fig. S2A and B). The top 25 genes are shown for a more refined reference.

Reactome 2022 and WikiPathway 2023 analyses predicted pathways up and downregulated in the livers of *mMgat2* and *HuMgat2* mice fed the CDAA-HFD. Extracellular matrix synthesis and degradation were upregulated (supplemental Fig. S3 and S4) that correlated with increased expression of *Mmp7/12* in both cohorts (supplemental Tables S1 and S2). Feeding *HuMgat2* mice the CDAA-HFD downregulated adipocyte differentiation and adipogenesis pathways, as well as leptin and adiponectin signaling (supplemental Fig. S4). A 60% reduction in *Lep* gene expression was observed in livers of *HuMgat2* mice (supplemental Table S1). IL-4/IL-10 signaling was upregulated in *mMgat2* mice due to CDAA-HFD-feeding and *Cxcr1* expression was induced (supplemental Table S3). *mMgat2* mice fed the CDAA-HFD were predicted to downregulate triglyceride synthesis and metabolism.

In response to elafibranor treatment, *mMgat2* and *HuMgat2* mice both upregulated pathways involved in triglyceride catabolism, w3, w6 fatty acid synthesis, mitochondrial fatty acid β-oxidation, and lipid transport (supplemental Fig. S3 and S4). Surprisingly, *HuMgat2* mice treated with elafibranor had increased expression of *Sptlc3* (3.7-fold) and *Fabp3* (2.9-fold) which are genes associated with MASH development (supplemental Table S1) (62, 63). We also observed elafibranor-dependent effects on the expression of the *Gabrg1* and *Gabrb2* γ-aminobutyric acid type A/B receptor subunits γ1 and β2 in both cohorts (supplemental Table S1). Both were downregulated in *HuMgat2* mice fed the CDAA-HFD but elevated upon elafibranor treatment (supplemental Table S1). While there was no effect on the expression of these genes in *mMgat2* mice fed the CDAA-HFD, treating with elafibranor treatment increased expression (supplemental Table S2).

Both cohorts showed upregulation of PPAR signaling in response to elafibranor treatment (supplemental ig. S2, C and D). PPARα genes that were induced included *Aqp3, Acot2, Fabp3, Fabp4*, and *Lepr* (supplemental Fig. S2, C and D), while PPARα/δ genes induced included *VLDLR*, *GADD45B*, and *ACC1/2* (supplemental Fig. S2, C and D).

Reactome 2022 predicated that elafibranor-treated *HuMgat2* mice harbored changes in bile acid salt transport (supplemental Fig. S3). Profiling a subset of bile acid transporters showed that elafibranor induced *MRP4* expression, while decreasing Ostβ and *Abst* expression in CDAA-HFD-fed mice (both cohorts), suggesting an elafibranor-dependent induction in bile acid efflux (supplemental Fig. S2E).

### Bile acid metabolism and composition are altered in livers of *HuMgat2* mice and restored by elafibranor treatment

Several bile acid species act as FXR ligands that induce the expression of FXR-dependent genes. We did see changes in the expression of a subset of FXR-dependent genes (*Vldl*, *Apoc3*, *Abcg5*, *cyp7b1*) that were statistically significant when comparing CDAA-HFD versus CDAA-HFD + ELA (supplemental Fig S2F). Thus, we determined the bile acid compositions of *mMgat2* and *HuMgat2* mice fed the various diets.

The BA profile was analyzed using BA indices that specify the BA composition, metabolism, hydrophilicity, and toxicity (supplemental Table S3). The advantage of BA indices is that they have lower inter- and intra-individual variability compared to net concentrations of BAs (64). In addition, BA indices may better reflect the risk of liver malfunction, especially when used as a potential diagnostic biomarker for cholestatic liver diseases that are highly associated with the severity of any chronic liver diseases, including MASLD/MASH (65).

**Fig. 11:**
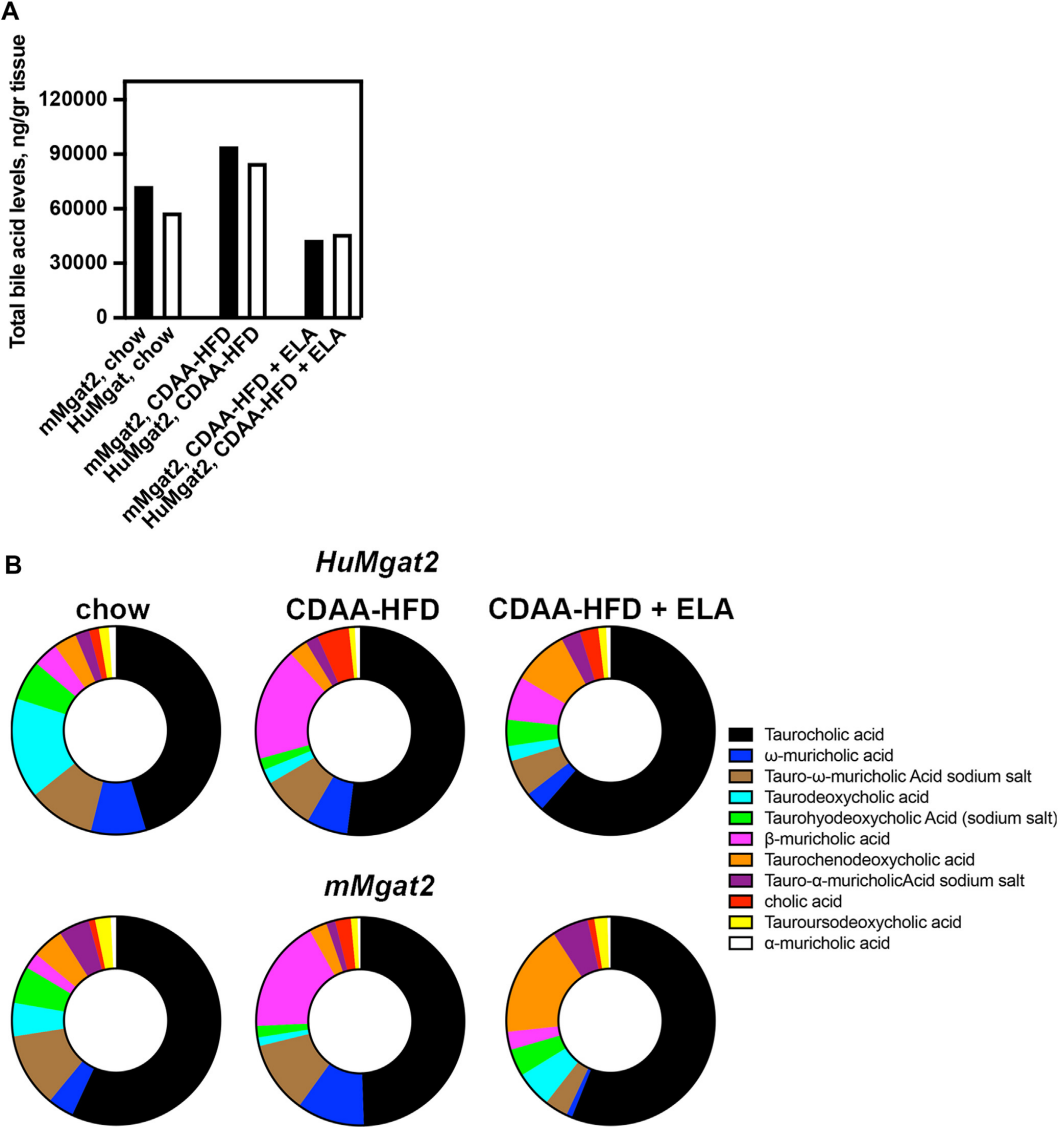
Livers of *HuMgat2* mice develop cholestasis due to increased accumulation of toxic bile acids. Bile acid levels were determined from three individual livers from *mMgat2* and *HuMgat2* mice fed the various diets. (A), total bile acid levels were the average values, (B), Percentage of top 11 bile acids comprising >95% of the total bile acid composition.

Both *mMgat2* and *HuMgat2* mice with MASH had highly increased BA levels in their livers that were markedly reduced by treatment of ELA (Fig. 11A). Primary BAs levels in these mice were increased and restored to normal by elafibranor (Fig. 11B). The ratio of primary versus secondary BAs indicates the degree of BA metabolism by the gut microbiome, as secondary BAs are generated by gut microbiome. There was a large induction in the primary:secondary ratios in *mMgat2* (5-fold) and *HuMgat2* (8-fold) mice with MASH (supplemental Table S3). The elevated ratio seen for livers of *mMgat2* mice was fully restored but to a lesser extent in the livers of *HuMgat2* mice.

Metabolism of BAs by sulfation or amidation may promote BA efflux through sinusoidal to blood efflux and canulicular to bile efflux, respectively. In mice, taurine dominates over glycine conjugation of BAs, but in humans’ glycine conjugation dominates over taurine conjugation. In *Mgat2* mice, the percentile of sulfated BAs was increased by HFD, which was not altered by ELA treatment. *HuMgat2* fed chow had a higher percentage of sulfated BAs that were not affected by feeding these mice the CDAA-HFD or treating with elafibranor. *mMgat2* mice fed the chow diet showed a higher degree of amidation that decreased upon feeding the CDAA-HFD, which was restored by elafibranor. *HuMgat2* mice while showing a similar trend, showed a lower ratio of amidation, especially following elafibranor treatment.

BA hydrophobicity is an important indicator of BA toxicity with less hydroxylated BA being more cytotoxic. *mMgat2* mice fed the CDAA-HFD had markedly reduced levels of mono-OH BA (LCA), which was restored upon elafibranor treatment to ∼25% of mice fed the chow diet. *HuMgat2* mice fed the chow diet had a much lower mono-OH BA ratio, which was increased by the CDAA-HFD ∼3-fold and further increased by ∼2–3 fold upon elafibranor treatment. The di-OH BAs including CDCA, UDCA, DCA, and HDCA, were reduced in mice fed the CDAA-HFD but restored by elafibranor treatment (Fig. 8D). The Tri-OH BAs, which include CA and MCA, which are most abundant BAs in mice, were increased in mice fed the CDA-HFD and restored to normal levels upon elafibranor treatment.

## Discussion

The number of individuals with MASLD is estimated at 20%–40% of the world’s population (1). This is most likely an underestimate based on the benign nature of the disease and the lack of early-stage diagnostics (2). MASH, the more severe form of the disease, is also continuing to rise at an alarming rate (66). A subpopulation of individuals with MASH will progress to cirrhosis and HCC if left untreated (67). Only a single therapeutic exists to treat MASLD/MASH and no diagnostics are currently on the market, thus there remains a critical unmet medical need for additional diagnostics and drug treatments.

### *HuMgat2* mice as a pre-clinical drug efficacy model for testing MOGAT2 inhibitors

Our data support the idea that humanized *HuMgat2* mice will serve as a pre-clinical model for testing MOGAT2 inhibitors for in vivo efficacy in treating MASH. *HuMgat2* mice fed the CDAA-HFD displayed metabolic and histological hallmarks of MASH with liver fibrosis. They responded properly to elafibranor treatment, as PPARα and PPARα/δ signaling was activated and drug treatment attenuated most MASH phenotypes. Possible differential liver cytokine profiles were observed between the cohorts, giving information as to potential efficacy biomarker identification for potential clinical trials. RNASeq transcriptome profiles and the pathways predicted to be regulated by diet significantly overlapped, and cholestasis was observed that correlated with expression changes in bile acid transporters, further supporting model efficacy.

### *MOGAT2* and *HuMgat2* mice, and their translatability to metabolic diseases

A study comparing RNASeq data from *ob/ob* mice fed an HFD with that of MASH patients shows a high degree of translatability (68). In one instance, bile acid metabolism was a common pathway affected. Bile acid transporters like *Slc51* *b/Ostβ*, *Abcb11/Bsep*, and *Oatp1a2* were differentially regulated. We observed the same trend in both cohorts fed the CDAA-HFD. A search of UniProt (https://www.uniprot.org/uniprotkb/Q3SYC2/entry) shows the presence of several *MOGAT2* genetic variants, but none are linked to MASLD/MASH. A recent genome-wide human exome ChIP study did uncover a genetic variant in *MOGAT2* (rs499972) linked to the accumulation of diabetes-associated metabolites (69, 70). Our preliminary data has shown that HuMgat2 mice fed a Western diet become obese, glucose intolerant, insulin resistant, and pre-diabetic (*J. Corbalan and J. Nickels, unpublished results*).

The relationship between loss in normal autophagic flux and MASH is well established, and defects were seen in our mice fed the CDAA-HFD (71). Very recently, Deng *et al.*, (72) used artificial learning models to search the GEO database (https://www.ncbi.nlm.nih.gov/geo/) looking for any relationship between defects in mitophagy and MASH. Several mitophagy-related processes were uncovered and small molecule drugs against specific related targets were designed. It has been shown in rats that gastric bypass lowers *MGAT2* and *DGAT2* expression and this correlates with reinitiation of autophagy (73).

### RNASeq analyses of HuMgat2 mice with MASH and response to elafibranor treatment

Several RNASeq studies have generated the transcriptomic profiles of mice with MASH treated with elafibranor (74, 75, 76). *ob*/*ob* mice fed a HFD activated PPAR signaling, fatty acid β-oxidation, and triglyceride catabolism. We also found these pathways were upregulated in the livers of *mMgat2* and *HuMgat2* mice. APOE∗3Leiden.CETP mice fed an HFD were shown to activate pathways that greatly overlapped with those of individuals with MASH (87%) (74). Elafibranor dampened inflammatory response and fibrosis gene expression. Tobol *et al.*, (75) saw an increase in PPAR-dependent gene expression that included *Fasn*, *Scd1*, and *Cd36* in DIO mice fed an AMYLN diet. We also observed expression changes in these genes. Pathway analysis suggested upregulation of PPAR signaling, biosynthesis of unsaturated fatty acids, oxidative phosphorylation, and fatty acid elongation.

*LEP* expression was reduced by 60% in *HuMgat2* mice fed the CDAA-HFD. A further search of the RNASeq data for *mMgat2* mice fed the CDAA-HFD found that *LEP* gene expression was decreased by 50%. Leptin is a satiety factor known to stimulate anorexigenic hormone secreting POMC neurons while inhibiting orexigenic hormone secreting AgRP neurons (77). It is secreted by adipose tissue in response to an increase in fat mass (78). *mMgat2* and *HuMgat2* mice fed the CDAA-HFD did not gain weight, so it may be that *LEP* expression was not triggered due to the lack of adipose tissue expansion.

### Bile acid transporter expression and bile acid composition is altered in *HuMgat2* with MASH

Bile acid transporter expression was altered in mice treated with elafibranor. Based on our gene expression predictions, *HuMgat2* mice with MASH would have accumulated toxic bile acids in their livers due to loss of export capability, with elafibranor treatment restoring efflux. *HuMgat2* mice had highly elevated total bile acid levels in their livers that were reduced by elafibranor treatment. Gut microbiota play an important part in maintaining a proper primary:secondary bile acid ratio. This ratio was altered in *HuMgat2* mice and restored with elafibranor treatment but not to the extent of that ratio seen for *mMgat2* mice. Reducing secondary BAs may provide a health benefit, as an increase in secondary BAs are associated with liver disease progression. Bile acid amination in *HuMgat2* mice although functional, was less efficient. Thus, while ELA was effective in promoting normal liver functions by promoting amidation but not by sulfation, HuMgat2 mice may have responded less efficiently than *mMgat2* mice regarding this beneficial effect. Finally, the fact that the ratio of 12α-OH/non 12α-OH BAs was not fully corrected in elafibranor-treated *HuMgat2* mice may suggest that these mice are less efficient at restoring the classical bile acid pathway despite a reduction in total bile acids.

### Is MOGAT2 a target for treating MASLD/MASH?

*mMgat2* mice fed a HFD have normal fat absorption yet contain markedly reduced hepatic triglycerides levels (79, 80). Mice treated with MOGAT2 inhibitors have shown the same results (18, 19). Treating healthy obese individuals with the MOGAT2 inhibitor BMS-963272 caused a downward trend in blood triglycerides after 15 days of treatment (21). Recent studies using BMS-963272 demonstrated its effectiveness in causing weight loss in mice and in healthy obese humans, while also increasing GLP1 levels, a robust satiety factor (21). The dual effects of MOGAT2 inhibitors on lowering triglycerides and causing weight loss has great potential in treating MASLD/MASH, as obesity is a major risk factor in driving the progression of MASLD/MASH (26, 81, 82).

### Limitations of the study

It must be pointed out that a potential limitation of our study is that only male mice were used. Sex differences are believed to have effects on metabolism (83) and body temperature, which could have effects on MASLD/MASH progression (84). Several studies have shown that diet-induced obesity and type two diabetes phenotypes are more severe in male rats (85) and ovariectomized mice (86), respectively.

Another limitation concerns the possible effect of HUR protein on increasing *hMOGAT2* pre-mRNA stability. HuR stabilizes *mMgat2* mRNA by regulating pre-mRNA intron spicing (22). Mice lacking intestinal *HUR* have lower *Mgat2* expression levels in the proximal jejunum than wild-type littermates, and have less protein (22). In our study, a human cDNA was used to express human *MOGAT2*, which lacked the introns found within the endogenous mouse *Mgat2* gene. If HUR stabilized *mMgat2* mRNA and thus MGAT2 protein, one might predict that a more severe phenotype would be seen in *mMgat2* mice. Supplemental Fig. S1C does indicate that under chow feeding conditions there is no difference in gene expression of the *HuMgat2* cDNA from the endogenous mouse promoter and the mouse chromosomal *MGAT2* gene.

## Data availability

All data are available upon request.

## Supplemental data

This article contains supplemental data.

## Conflict of interests

The author is an Editorial Board Member/Editor-in-Chief/Associate Editor/Guest Editor for [Journal name] and was not involved in the editorial review or the decision to publish this article.

The authors declare the following financial interests/personal relationships which may be considered as potential competing interests: The corresponding author is an Editorial Board Member of the Journal of Lipid Research and was not involved in the editorial review or decision to publish this article JC, PJ, KKK, GG, and JN are either present or were past employees of Genesis Global Group.

## Supplementary data

Supplemental Figures and Legends

Cyagen HuMgat2 Project 12.2023
