# Supplementary material for: Humanized monoacylglycerol acyltransferase 2 mice develop metabolic dysfunction-associated steatohepatitis
Source: J Lipid Res. 2024 Nov 5;65(12):100695. doi: 10.1016/j.jlr.2024.100695 (PMC11648239; doi:10.1016/j.jlr.2024.100695)

Supplemental full-length westerns

1. **Supplemental Full-Length westerns blots used for western figures**

pg.1. pp62
pg.2. Ubiquitinated protein aggregates
pg.3. LC3-I and LC3-II
pg.4. COL1A1
pg.5. COL3A1
pg.6. SMA
pg.7. Caspase 3
pg.8. PARP1

Supplemental pp62 western blots

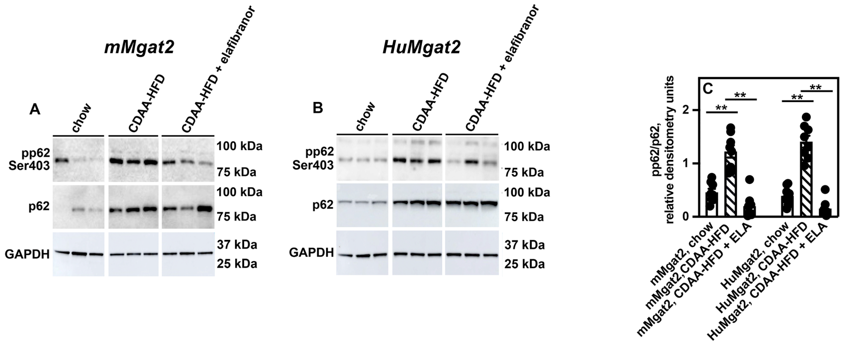

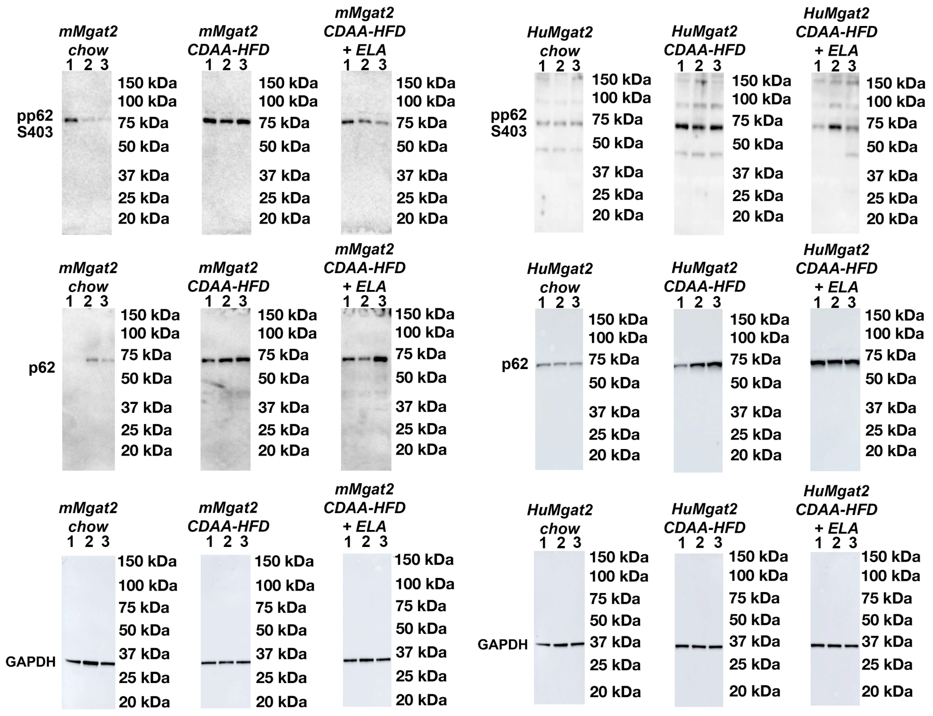

Supplemental Ub western

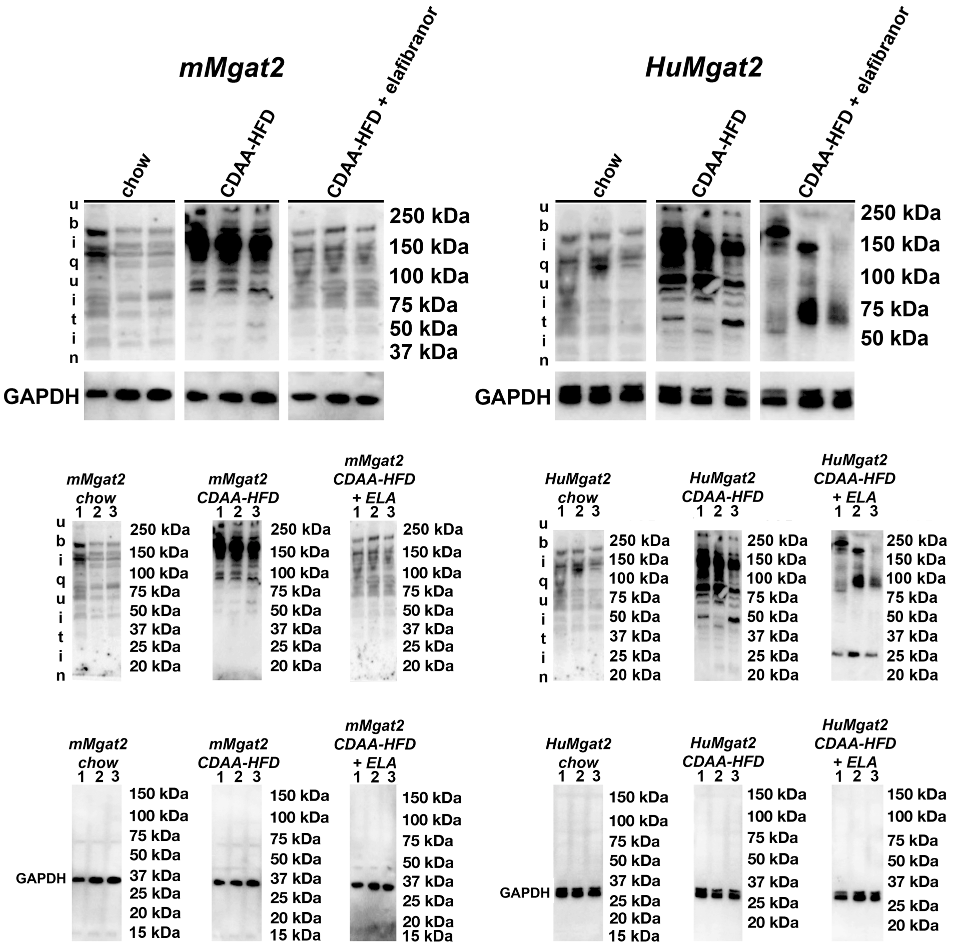

Supplemental LC3-I/LC3-II western

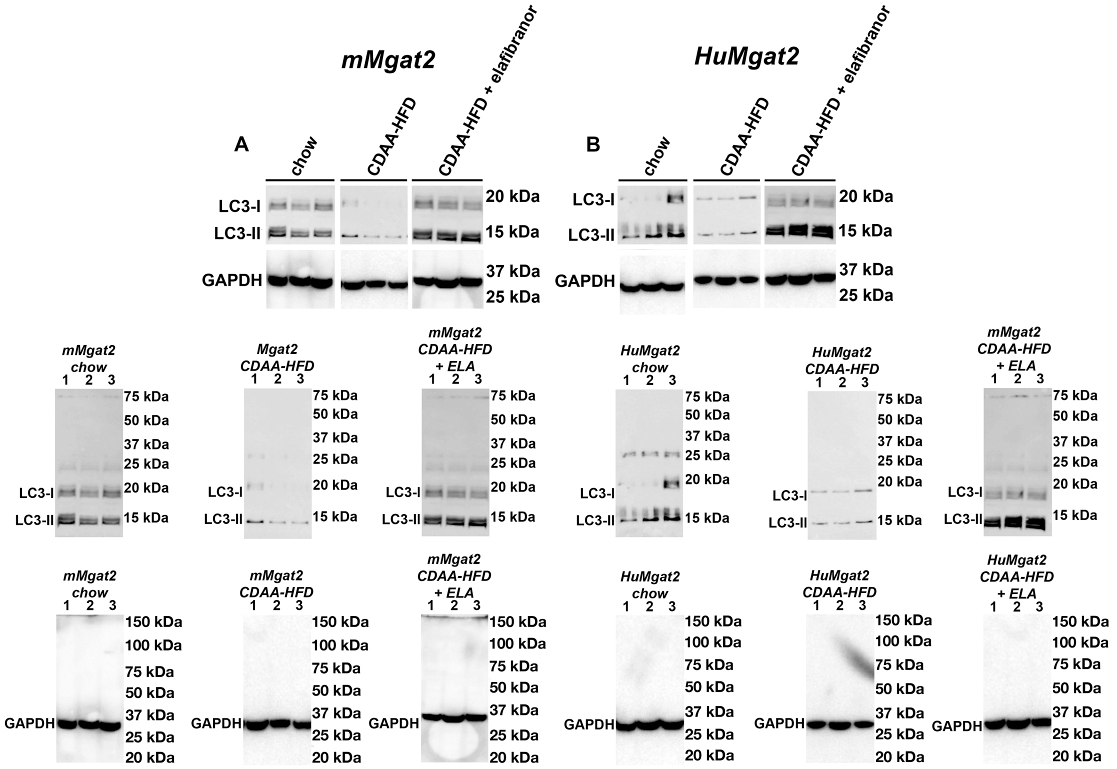

Supplemental COL1A1 western pg. 4

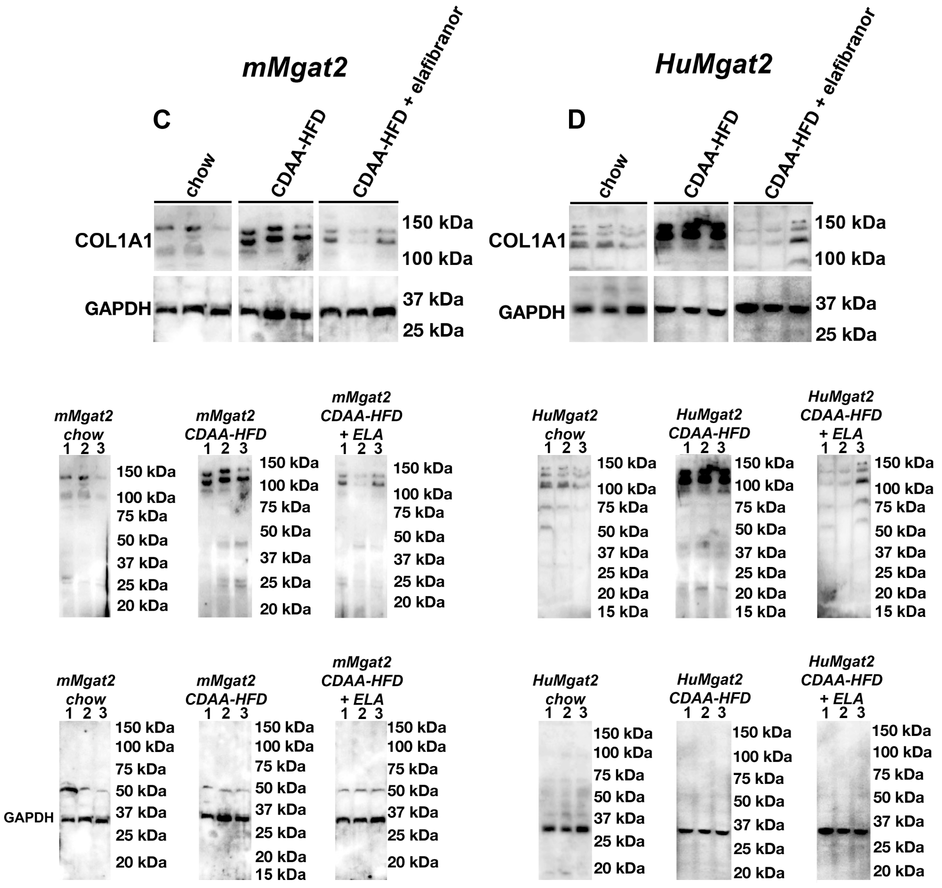

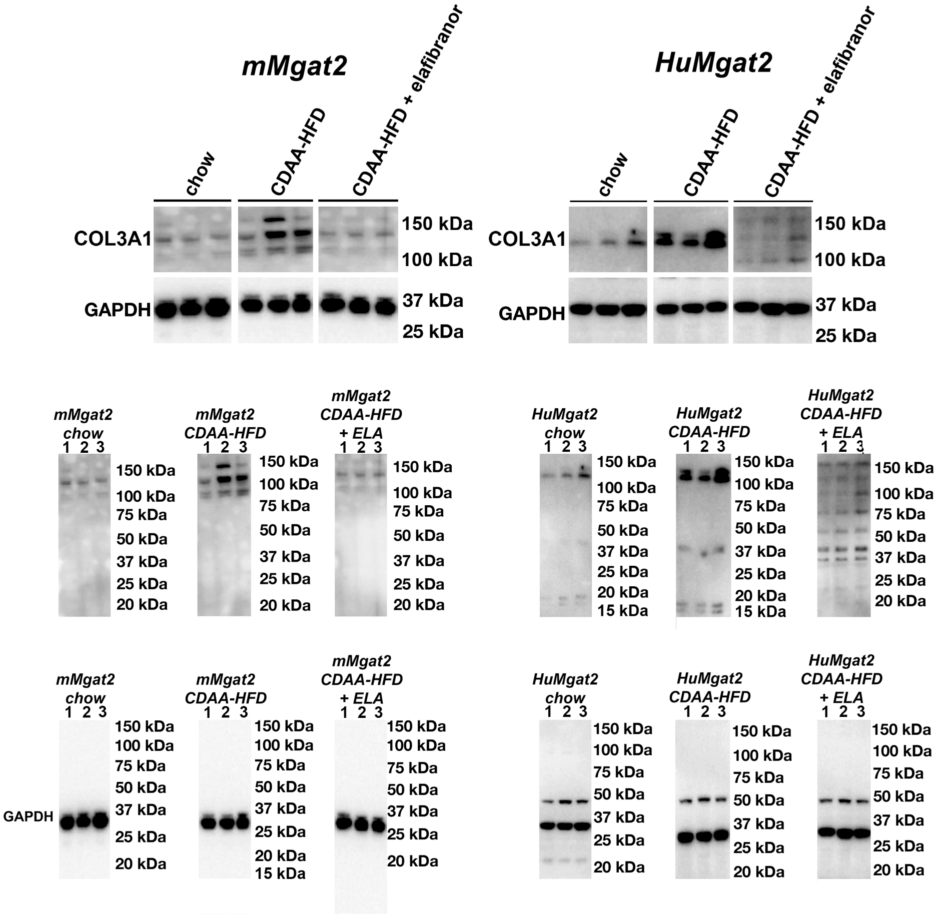
mMgat2
HuMgat2
chow
CDAA-HFD
CDAA-HFD + elafibranor
COL3A1
GAPDH
150 kDa
100 kDa
37 kDa
25 kDa
mMgat2 chow
mMgat2 CDAA-HFD
mMgat2 CDAA-HFD + ELA
HuMgat2 chow
HuMgat2 CDAA-HFD
HuMgat2 CDAA-HFD + ELA
1 2 3
75 kDa
50 kDa
20 kDa
15 kDa

Supplemental SMA western

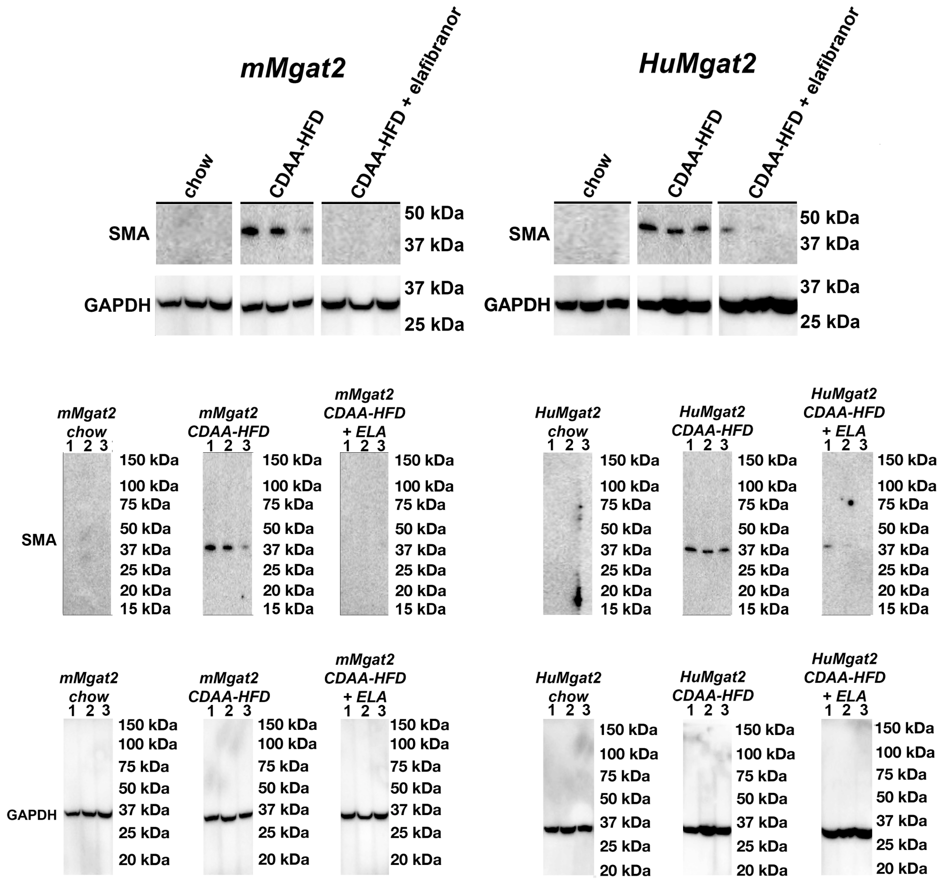

Supplemental Caspase 3 western

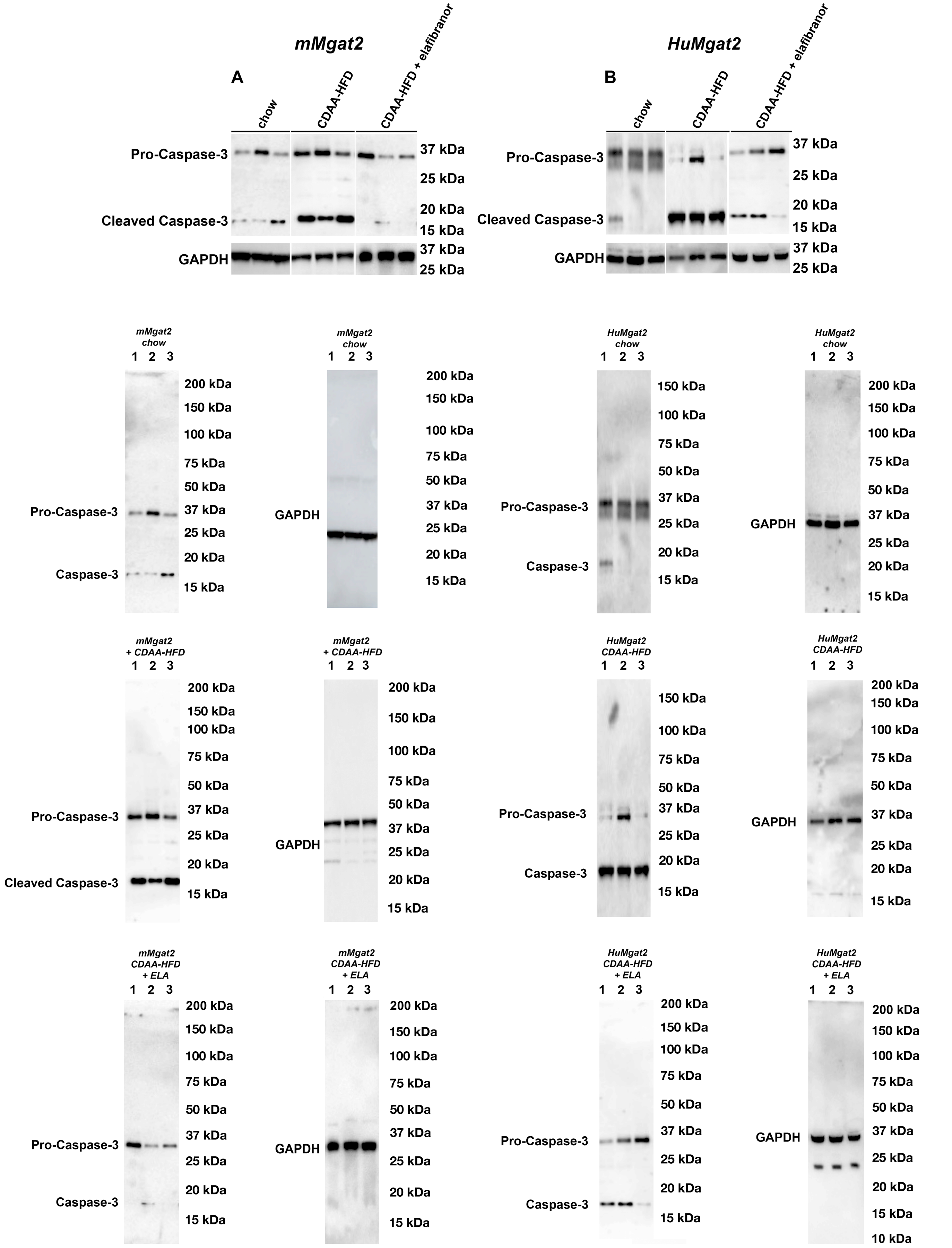

Supplemental PARP1 western

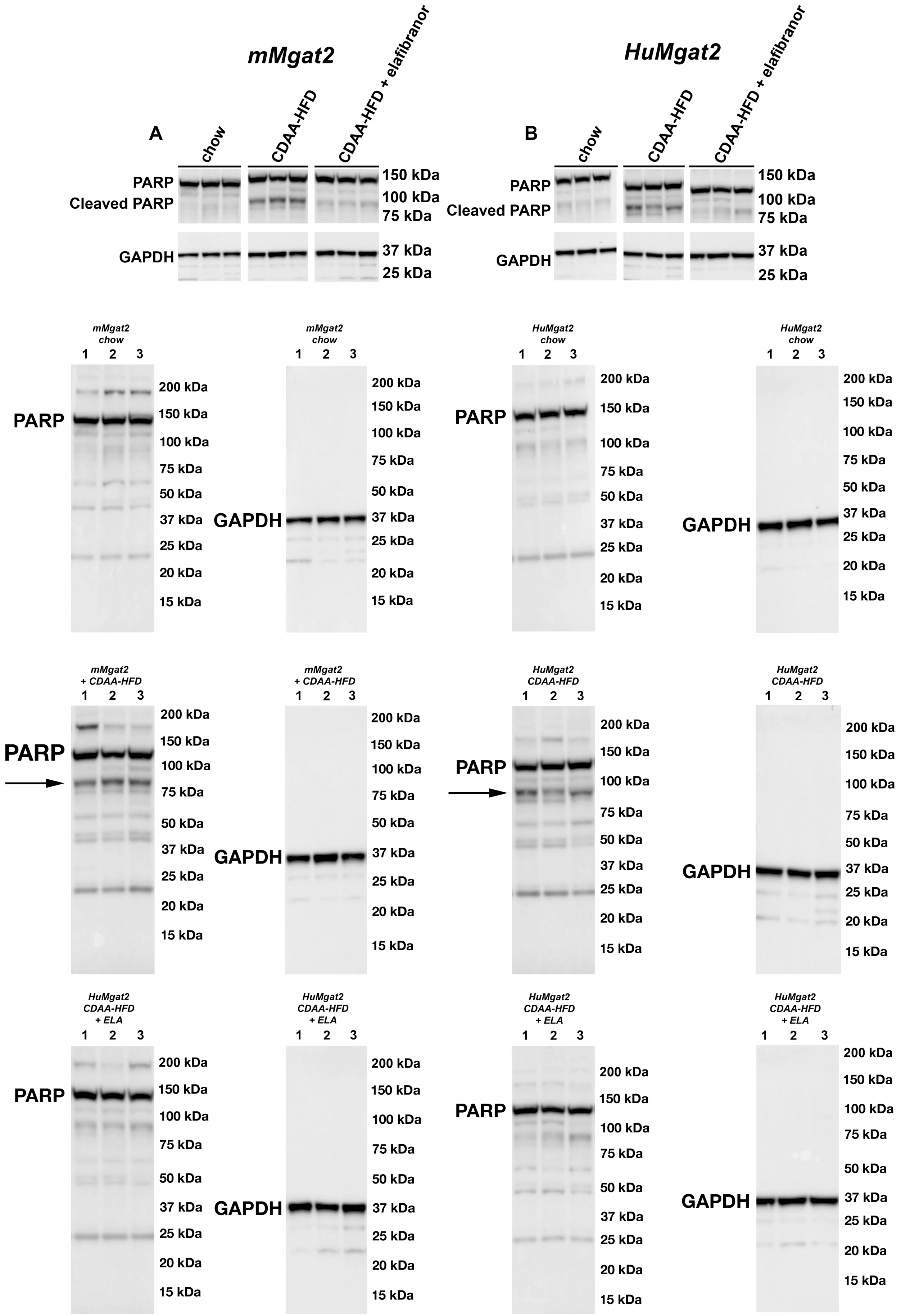

Supplement: Supplemental data [file mmc1.pdf]
