# Supplementary material for: Humanized monoacylglycerol acyltransferase 2 mice develop metabolic dysfunction-associated steatohepatitis
Source: J Lipid Res. 2024 Nov 5;65(12):100695. doi: 10.1016/j.jlr.2024.100695 (PMC11648239; doi:10.1016/j.jlr.2024.100695)
Supplement: Supplemental Tables [file mmc2.pdf]

Table S1. *Top 10 genes up and down regulated in HuMgat2 mice*			
Gene	Name	Log_2 Fold Change	Reference
**Top 10 genes up regulated *in HuMgat2 mice fed the CDAA-HFD vs. HuMgat2 mice fed the chow diet***			
GPNMB	glycoprotein NMB	9.79	(1)
GLDN	gliomedin	9.79	(2)
VMN2R3	vomeronasal receptor 3, subfamily 2	9.41	(3)
H19	lncRNA	8.47	(4)
MMP12	matrix metalloproteinase 12	8.46	(5)
ASZ1	ankyrin repeat, SAM and basic leucine zipper domain-containing protein 1	8.07	(6)
TRIM29	tripartite motif protein 29	7.67	(7)
VSIG8	v-set and immunoglobulin domain containing 8	7.29	(8)
FBN2	fibrillin 2	7.29	(9)
MMP7	matrix metalloproteinase 7	7.15	(5)
**Top 10 genes down regulated *in HuMgat2 mice fed the CDAA-HFD vs. HuMgat2 mice fed the chow diet***			
CYP2C53-ps	cytochrome P450, family 2, subfamily c, polypeptide 53, pseudogene	-9.88	(10)
TRHDE	thyrotropin Releasing Hormone Degrading Enzyme	-7.09	(11)
GTPBP4-ps4	GTP binding protein, *pseudogene 4*	-6.12	(12)
SULT3A1	sulfotransferase family 3A, member 1	-5.92	(13)
CIDEA	cell death inducing DFFA effector A	-5.87	(14)
OBP2a	odorant binding protein 2A	-5.85	(15)
GTPBP4-ps1	GTP binding protein, *pseudogene 4*	-5.67	(12)
Gm49012	-	-5.64	
LEP	leptin	-5.61	(16)

CYP2C29	cytochrome P450, family 2, subfamily c, polypeptide 29	-5.58	(17)
**Top 10 genes up regulated *in HuMgat2 mice fed the CDAA-HFD + elafibranor vs. HuMgat2 mice fed the CDAA-HFD diet***			
OTOP1	otopetrin	13.44	(18, 19)
SPTLC3	serine palmitoyltransferase long chain base subunit 3	12.4	(20)
GABRB2	g-aminobutyric acid type B receptor subunit 2	11.70	(21)
AQP3	aquaporin 3	10.99	(22)
Gm5087	-		
CIDEA	cell death inducing DFFA effector A	10.56	(15)
GABRG1	g-aminobutyric acid type A receptor subunit 1	9.91	(23)
FABP3	fatty acid binding protein 3	9.26	(24)
AU01809	-	9.24	
PNLIPRP1	pancreatic lipase related protein 1	9.23	(25)
**Top 10 genes down regulated *in HuMgat2 mice fed the CDAA-HFD + elafibranor vs. HuMgat2 mice fed the CDAA-HFD diet***			
GLDN	gliomedin	-8.73	(2)
LRTM1	leucine rich repeats and transmembrane domains 1	-8.01	(26)
Gm28301	-	-6.97	
NTRK2	TRK neurotrophic receptor tyrosine kinase 2	-6.83	(27)
C6300044B11Rik		-6.28	
CLEH2	C-type lectin domain family 2, member H	-6.23	
RTL1	retrotransposon Gag-like 1	-6.21	(28)
B8300012L14Rik	-	-6.17	
Gm24598	mortality factor like pseudogene	-6.16	(29)
SERPINA12	serine family A member 12	-6.16	(30)

References

1. Saade, M., Araujo de Souza, G., Scavone, C., andKinoshita, P. F. (2021) The Role of GPNMB in Inflammation Front Immunol **12**, 674739 10.3389/fimmu.2021.674739

2. Eshed, Y., Feinberg, K., Poliak, S., Sabanay, H., Sarig-Nadir, O., Spiegel, I. *et al.* (2005) Gliomedin mediates Schwann cell-axon interaction and the molecular assembly of the nodes of Ranvier Neuron **47**, 215-229 10.1016/j.neuron.2005.06.026

3. Francia, S., Silvotti, L., Ghirardi, F., Catzeflis, F., Percudani, R., andTirindelli, R. (2014) Evolution of spatially coexpressed families of type-2 vomeronasal receptors in rodents Genome Biol Evol **7**, 272-285 10.1093/gbe/evu283

4. Gabory, A., Ripoche, M. A., Le Digarcher, A., Watrin, F., Ziyyat, A., Forne, T. *et al.* (2009) H19 acts as a trans regulator of the imprinted gene network controlling growth in mice Development **136**, 3413-3421 10.1242/dev.036061

5. Okazaki, I., Noro, T., Tsutsui, N., Yamanouchi, E., Kuroda, H., Nakano, M. *et al.* (2014) Fibrogenesis and Carcinogenesis in Nonalcoholic Steatohepatitis (NASH): Involvement of Matrix Metalloproteinases (MMPs) and Tissue Inhibitors of Metalloproteinase (TIMPs) Cancers (Basel) **6**, 1220-1255 10.3390/cancers6031220

6. van der Harst, P., andVerweij, N. (2018) Identification of 64 Novel Genetic Loci Provides an Expanded View on the Genetic Architecture of Coronary Artery Disease Circ Res **122**, 433-443 10.1161/CIRCRESAHA.117.312086

7. Xu, M., Hu, J., Zhou, B., Zhong, Y., Lin, N., andXu, R. (2019) TRIM29 prevents hepatocellular carcinoma progression by inhibiting Wnt/beta-catenin signaling pathway Acta Biochim Biophys Sin (Shanghai) **51**, 68-77 10.1093/abbs/gmy151

8. Cao, S., Li, H., Xin, J., Jin, Z., Zhang, Z., Li, J. *et al.* (2023) Identification of genetic profile and biomarkers involved in acute respiratory distress syndrome Intensive Care Med 10.1007/s00134-023-07248-9

9. Huang, Y., Liangpunsakul, S., Rudraiah, S., Ma, J., Keshipeddy, S. K., Wright, D. *et al.* (2023) HMGB2 is a potential diagnostic marker and therapeutic target for liver fibrosis and cirrhosis Hepatol Commun **7**, 10.1097/HC9.0000000000000299

10. Pfohl, M., Marques, E., Auclair, A., Barlock, B., Jamwal, R., Goedken, M. *et al.* (2021) An 'Omics Approach to Unraveling the Paradoxical Effect of Diet on Perfluorooctanesulfonic Acid (PFOS) and Perfluorononanoic Acid (PFNA)-Induced Hepatic Steatosis Toxicol Sci **180**, 277-294 10.1093/toxsci/kfaa172

11. Zhang, F., Zhang, Z., Li, Y., Sun, Y., Zhou, X., Chen, X. *et al.* (2022) Integrated Bioinformatics Analysis Identifies Robust Biomarkers and Its Correlation With Immune Microenvironment in Nonalcoholic Fatty Liver Disease Front Genet **13**, 942153 10.3389/fgene.2022.942153

12. Zhou, Q., Yin, Y., Yu, M., Gao, D., Sun, J., Yang, Z. *et al.* (2022) GTPBP4 promotes hepatocellular carcinoma progression and metastasis via the PKM2 dependent glucose metabolism Redox Biol **56**, 102458 10.1016/j.redox.2022.102458

13. Yalcin, E. B., More, V., Neira, K. L., Lu, Z. J., Cherrington, N. J., Slitt, A. L. *et al.* (2013) Downregulation of sulfotransferase expression and activity in diseased human livers Drug Metab Dispos **41**, 1642-1650 10.1124/dmd.113.050930

14. Zhou, L., Xu, L., Ye, J., Li, D., Wang, W., Li, X. *et al.* (2012) Cidea promotes hepatic steatosis by sensing dietary fatty acids Hepatology **56**, 95-107 10.1002/hep.25611

15. Zhou, Z., Yon Toh, S., Chen, Z., Guo, K., Ng, C. P., Ponniah, S. *et al.* (2003) Cidea-deficient mice have lean phenotype and are resistant to obesity Nat Genet **35**, 49-56 10.1038/ng1225

16. Polyzos, S. A., Kountouras, J., andMantzoros, C. S. (2015) Leptin in nonalcoholic fatty liver disease: a narrative review Metabolism **64**, 60-78 10.1016/j.metabol.2014.10.012

17. Li, H., Clarke, J. D., Dzierlenga, A. L., Bear, J., Goedken, M. J., andCherrington, N. J. (2017) In vivo cytochrome P450 activity alterations in diabetic nonalcoholic steatohepatitis mice J Biochem Mol Toxicol **31**, 10.1002/jbt.21840

18. Wang, G. X., Cho, K. W., Uhm, M., Hu, C. R., Li, S., Cozacov, Z. *et al.* (2014) Otopetrin 1 protects mice from obesity-associated metabolic dysfunction through attenuating adipose tissue inflammation Diabetes **63**, 1340-1352 10.2337/db13-1139

19. Tu, Y. H., Liu, N., Xiao, C., Gavrilova, O., andReitman, M. L. (2023) Loss of Otopetrin 1 affects thermoregulation during fasting in mice PLoS One **18**, e0292610 10.1371/journal.pone.0292610

20. Ijuin, S., Oda, K., Mawatari, S., Taniyama, O., Toyodome, A., Sakae, H. *et al.* (2022) Serine palmitoyltransferase long chain subunit 3 is associated with hepatocellular carcinoma in patients with NAFLD Mol Clin Oncol **16**, 55 10.3892/mco.2021.2488
21. Taneera, J., Jin, Z., Jin, Y., Muhammed, S. J., Zhang, E., Lang, S. *et al.* (2012) gamma-Aminobutyric acid (GABA) signalling in human pancreatic islets is altered in type 2 diabetes Diabetologia **55**, 1985-1994 10.1007/s00125-012-2548-7
22. Tardelli, M., Claudel, T., Bruschi, F. V., Moreno-Viedma, V., andTrauner, M. (2017) Adiponectin regulates AQP3 via PPARalpha in human hepatic stellate cells Biochem Biophys Res Commun **490**, 51-54 10.1016/j.bbrc.2017.06.009
23. Mohamad, F. H., Mohamad Jamali, M. A., andChe Has, A. T. (2023) Structure-function Studies of GABA (A) Receptors and Related computer-aided Studies J Mol Neurosci **73**, 804-817 10.1007/s12031-023-02158-3
24. Storch, J., andCorsico, B. (2023) The Multifunctional Family of Mammalian Fatty Acid-Binding Proteins Annu Rev Nutr **43**, 25-54 10.1146/annurev-nutr-062220-112240
25. Wagner, F., Ruf, I., Lehmann, T., Hofmann, R., Ortmann, S., Schiffmann, C. *et al.* (2022) Reconstruction of evolutionary changes in fat and toxin consumption reveals associations with gene losses in mammals: A case study for the lipase inhibitor PNLIPRP1 and the xenobiotic receptor NR1I3 J Evol Biol **35**, 225-239 10.1111/jeb.13970
26. Li, H. K., Zhou, Y., Ding, J., Xiong, L., Shi, Y. X., He, Y. J. *et al.* (2020) LRTM1 promotes the differentiation of myoblast cells by negatively regulating the FGFR1 signaling pathway Exp Cell Res **396**, 112237 10.1016/j.yexcr.2020.112237
27. Gray, J., Yeo, G., Hung, C., Keogh, J., Clayton, P., Banerjee, K. *et al.* (2007) Functional characterization of human NTRK2 mutations identified in patients with severe early-onset obesity Int J Obes (Lond) **31**, 359-364 10.1038/sj.ijo.0803390
28. Shiura, H., Kitazawa, M., Ishino, F., andKaneko-Ishino, T. (2023) Roles of retrovirus-derived PEG10 and PEG11/RTL1 in mammalian development and evolution and their involvement in human disease Front Cell Dev Biol **11**, 1273638 10.3389/fcell.2023.1273638

29. Breitfeld, J., Kehr, S., Muller, L., Stadler, P. F., Bottcher, Y., Bluher, M. *et al.* (2020) Developmentally Driven Changes in Adipogenesis in Different Fat Depots Are Related to Obesity Front Endocrinol (Lausanne) **11**, 138 10.3389/fendo.2020.00138

30. Arab, J. P., Cabrera, D., Sehrawat, T. S., Jalan-Sakrikar, N., Verma, V. K., Simonetto, D. *et al.* (2020) Hepatic stellate cell activation promotes alcohol-induced steatohepatitis through Igfbp3 and SerpinA12 J Hepatol **73**, 149-160 10.1016/j.jhep.2020.02.005

Table S2. *Top 10 genes up and down regulated genes in mMgat2 mice fed various diets*

Gene	Name	Log_2 Fold Change	Reference
**Top 10 genes up regulated in *mMgat2* mice fed the CDAA-HFD *vs. mMgat2* mice fed the chow diet**			
GPNMB	glycoprotein NMB	11.8	(1)
VMN2R3	vomeronasal receptor 3, subfamily 2	10.2	(2)
MMP7	matrix metalloproteinase 7	10.1	(3)
CFAP44	-	9.56	-
GLDN	gliomedin	9.27	(4)
MMP12	matrix metalloproteinase 12	9.11	(3)
FBN2	fibrillin 2	8.59	(5) (6)
DCLK1	doublecortin-like kinase 1	8.35	(7)
LRRC55	leucine-rich repeat-containing protein 8D	8.34	(8)
VSIG8	v-set and immunoglobulin domain containing 8	7.97	(9)
***Top 10 Genes down regulated* in *mMgat2* mice fed the CDAA-HFD *vs. mMgat2* mice fed the chow diet**			
CYP2B10	cytochrome p450, subfamily 2, polypeptide 10	-11.3	(10)
TRHDE	thyrotropin Releasing Hormone Degrading Enzyme	-9.41	(11)
CYP2C53-ps	cytochrome P450, family 2, subfamily c, polypeptide 53, pseudogene	-8.43	(12)
Gm49012	-	-8.35	-
Gm31121	-	-8.13	-
Cyp2c29	cytochrome P450, family 2, subfamily c, polypeptide 29	-6.67	(12)
MOXD1	monooxygenase DBH like 1	-6.63	(13)
R3HDML	R3H domain containing-like protein	-6.35	(14)
OBP2A	odorant binding protein 2A	-6.17	(15)
DMRT1A	DMRT-like family A1	-5.91	(16)

**Top 10 genes up regulated in *mMgat2* mice fed the CDAA-HFD + elafibranor *vs. mMgat2* mice fed the *CDAA-HFD* diet**			
SPTLC3	serine palmitoyltransferase long chain base subunit 3	14.6	(17)
OTOP1	otopetrin 1	11.8	(18)
GABRB2	g-aminobutyric acid type B receptor subunit 2	11.8	(19)
SNN1G	sodium channel epithelial 1 sybunit g	11.8	(20)
AQP3	aquaporin 3	11.8	(21)
CIDEA	cell death inducing DFFA effector A	11.4	(22)
GABRG1	g-aminobutyric acid type A receptor subunit 1	11.4	(23)
CXCR1	CXC motif chemokine receptor 1	11.1	(24)
ACOT3	acyl-CoA thioesterase 3	10.8	(25)
FABP3	fatty acid binding protein	10.4	(26)
**Top 10 genes down regulated in *mMgat2* mice fed the CDAA-HFD + elafibranor *vs. mMgat2* mice fed the *CDAA-HFD* diet**			
HSD3B5	hydroxy-d-5-steroid dehydrogenase, 3b	-11.2	(27)
Gm11341	-	-10.5	-
URAD	(2-Oxo-4-Hydroxy-4-Carboxy-5-) decarboxylase	-10.3	(28)
SERPINA12	serpin family A member 12	-10.0	(29)
LRTM1	leucine rich repeats and transmembrane domains 1	-9.7	(30)
Gm11340	-	-9.0	
CYP2C53-ps	cytochrome P450, family 2, subfamily c, polypeptide 53-pseudogene	-8.9	(31)
Gm53019	-	-8.7	-
Gm12718	-	-8.6	-
CYP2C50	cytochrome P450, family 2, subfamily c, polypeptide 50	-8.6	(32)

References

1. Saade, M., Araujo de Souza, G., Scavone, C., andKinoshita, P. F. (2021) The Role of GPNMB in Inflammation Front Immunol **12**, 674739 10.3389/fimmu.2021.674739
2. Dinka, H., Le, M. T., Ha, H., Cho, H., Choi, M. K., Choi, H. *et al.* (2016) Analysis of the vomeronasal receptor repertoire, expression and allelic diversity in swine Genomics **107**, 208-215 10.1016/j.ygeno.2015.10.003
3. Okazaki, I., Noro, T., Tsutsui, N., Yamanouchi, E., Kuroda, H., Nakano, M. *et al.* (2014) Fibrogenesis and Carcinogenesis in Nonalcoholic Steatohepatitis (NASH): Involvement of Matrix Metalloproteinases (MMPs) and Tissue Inhibitors of Metalloproteinase (TIMPs) Cancers (Basel) **6**, 1220-1255 10.3390/cancers6031220
4. Eshed, Y., Feinberg, K., Poliak, S., Sabanay, H., Sarig-Nadir, O., Spiegel, I. *et al.* (2005) Gliomedin mediates Schwann cell-axon interaction and the molecular assembly of the nodes of Ranvier Neuron **47**, 215-229 10.1016/j.neuron.2005.06.026
5. Huang, Y., Liangpunsakul, S., Rudraiah, S., Ma, J., Keshipeddy, S. K., Wright, D. *et al.* (2023) HMGB2 is a potential diagnostic marker and therapeutic target for liver fibrosis and cirrhosis Hepatol Commun **7**, 10.1097/HC9.0000000000000299
6. Kanta, J. (2016) Elastin in the Liver Front Physiol **7**, 491 10.3389/fphys.2016.00491
7. Ding, L., Weygant, N., Ding, C., Lai, Y., andLi, H. (2023) DCLK1 and tuft cells: Immune-related functions and implications for cancer immunotherapy Crit Rev Oncol Hematol **191**, 104118 10.1016/j.critrevonc.2023.104118
8. Gonzalez-Perez, V., Zhou, Y., Ciorba, M. A., andLingle, C. J. (2022) The LRRC family of BK channel regulatory subunits: potential roles in health and disease J Physiol **600**, 1357-1371 10.1113/JP281952
9. Cao, S., Li, H., Xin, J., Jin, Z., Zhang, Z., Li, J. *et al.* (2024) Identification of genetic profile and biomarkers involved in acute respiratory distress syndrome Intensive Care Med **50**, 46-55 10.1007/s00134-023-07248-9
10. Heintz, M. M., Kumar, R., Rutledge, M. M., andBaldwin, W. S. (2019) Cyp2b-null male mice are susceptible to diet-induced obesity and perturbations in lipid homeostasis J Nutr Biochem **70**, 125-137 10.1016/j.jnutbio.2019.05.004

11. Zhang, F., Zhang, Z., Li, Y., Sun, Y., Zhou, X., Chen, X. *et al.* (2022) Integrated Bioinformatics Analysis Identifies Robust Biomarkers and Its Correlation With Immune Microenvironment in Nonalcoholic Fatty Liver Disease Front Genet **13**, 942153 10.3389/fgene.2022.942153

12. Li, R., Grimm, S. A., Mav, D., Gu, H., Djukovic, D., Shah, R. *et al.* (2018) Transcriptome and DNA Methylome Analysis in a Mouse Model of Diet-Induced Obesity Predicts Increased Risk of Colorectal Cancer Cell Rep **22**, 624-637 10.1016/j.celrep.2017.12.071

13. Ryaboshapkina, M., andHammar, M. (2017) Human hepatic gene expression signature of non-alcoholic fatty liver disease progression, a meta-analysis Sci Rep **7**, 12361 10.1038/s41598-017-10930-w

14. Sakamoto, K., Furuichi, Y., Yamamoto, M., Takahashi, M., Akimoto, Y., Ishikawa, T. *et al.* (2019) R3hdml regulates satellite cell proliferation and differentiation EMBO Rep **20**, e47957 10.15252/embr.201947957

15. Ekim Kocabey, A., andSchneiter, R. (2023) Human lipocalins bind and export fatty acids through the secretory pathway of yeast cells Front Microbiol **14**, 1309024 10.3389/fmicb.2023.1309024

16. Blanc, V., Riordan, J. D., Soleymanjahi, S., Nadeau, J. H., Nalbantoglu, I., Xie, Y. *et al.* (2021) Apobec1 complementation factor overexpression promotes hepatic steatosis, fibrosis, and hepatocellular cancer J Clin Invest **131**, 10.1172/JCI138699

17. Ijuin, S., Oda, K., Mawatari, S., Taniyama, O., Toyodome, A., Sakae, H. *et al.* (2022) Serine palmitoyltransferase long chain subunit 3 is associated with hepatocellular carcinoma in patients with NAFLD Mol Clin Oncol **16**, 55 10.3892/mco.2021.2488

18. Tu, Y. H., Liu, N., Xiao, C., Gavrilova, O., andReitman, M. L. (2023) Loss of Otopetrin 1 affects thermoregulation during fasting in mice PLoS One **18**, e0292610 10.1371/journal.pone.0292610

19. Ghit, A., Assal, D., Al-Shami, A. S., andHussein, D. E. E. (2021) GABA(A) receptors: structure, function, pharmacology, and related disorders J Genet Eng Biotechnol **19**, 123 10.1186/s43141-021-00224-0

20. Mares, S., Filipovsky, J., Vlkova, K., Pesta, M., Cerna, V., Hrabak, J. *et al.* (2021) A novel nonsense mutation in the beta-subunit of the epithelial sodium channel causing Liddle syndrome Blood Press **30**, 291-299 10.1080/08037051.2021.1942785

21. Tardelli, M., Claudel, T., Bruschi, F. V., Moreno-Viedma, V., andTrauner, M. (2017) Adiponectin regulates AQP3 via PPARalpha in human hepatic stellate cells Biochem Biophys Res Commun **490**, 51-54 10.1016/j.bbrc.2017.06.009

22. Zhou, Z., Yon Toh, S., Chen, Z., Guo, K., Ng, C. P., Ponniah, S. *et al.* (2003) Cidea-deficient mice have lean phenotype and are resistant to obesity Nat Genet **35**, 49-56 10.1038/ng1225

23. Rohbeck, E., Niersmann, C., Kohrer, K., Wachtmeister, T., Roden, M., Eckel, J. *et al.* (2023) Positive allosteric GABA(A) receptor modulation counteracts lipotoxicity-induced gene expression changes in hepatocytes in vitro Front Physiol **14**, 1106075 10.3389/fphys.2023.1106075

24. Pan, X., Chiwanda Kaminga, A., Liu, A., Wen, S. W., Chen, J., andLuo, J. (2020) Chemokines in Non-alcoholic Fatty Liver Disease: A Systematic Review and Network Meta-Analysis Front Immunol **11**, 1802 10.3389/fimmu.2020.01802

25. Regnier, M., Polizzi, A., Smati, S., Lukowicz, C., Fougerat, A., Lippi, Y. *et al.* (2020) Hepatocyte-specific deletion of Pparalpha promotes NAFLD in the context of obesity Sci Rep **10**, 6489 10.1038/s41598-020-63579-3

26. Storch, J., andCorsico, B. (2023) The Multifunctional Family of Mammalian Fatty Acid-Binding Proteins Annu Rev Nutr **43**, 25-54 10.1146/annurev-nutr-062220-112240

27. Guillen, N., Navarro, M. A., Arnal, C., Noone, E., Arbones-Mainar, J. M., Acin, S. *et al.* (2009) Microarray analysis of hepatic gene expression identifies new genes involved in steatotic liver Physiol Genomics **37**, 187-198 10.1152/physiolgenomics.90339.2008

28. Khan, S. A., Wollaston-Hayden, E. E., Markowski, T. W., Higgins, L., andMashek, D. G. (2015) Quantitative analysis of the murine lipid droplet-associated proteome during diet-induced hepatic steatosis J Lipid Res **56**, 2260-2272 10.1194/jlr.M056812

29. Arab, J. P., Cabrera, D., Sehrawat, T. S., Jalan-Sakrikar, N., Verma, V. K., Simonetto, D. *et al.* (2020) Hepatic stellate cell activation promotes alcohol-induced steatohepatitis through Igfbp3 and SerpinA12 J Hepatol **73**, 149-160 10.1016/j.jhep.2020.02.005

30. Li, H. K., Zhou, Y., Ding, J., Xiong, L., Shi, Y. X., He, Y. J. *et al.* (2020) LRTM1 promotes the differentiation of myoblast cells by negatively regulating the FGFR1 signaling pathway Exp Cell Res **396**, 112237 10.1016/j.yexcr.2020.112237

31. Pfohl, M., Marques, E., Auclair, A., Barlock, B., Jamwal, R., Goedken, M. *et al.* (2021) An 'Omics Approach to Unraveling the Paradoxical Effect of Diet on Perfluorooctanesulfonic Acid (PFOS) and

Perfluorononanoic Acid (PFNA)-Induced Hepatic Steatosis Toxicol Sci **180**, 277-294 10.1093/toxsci/kfaa172

32. Jamwal, R., andBarlock, B. J. (2020) Nonalcoholic Fatty Liver Disease (NAFLD) and Hepatic Cytochrome P450 (CYP) Enzymes Pharmaceuticals (Basel) **13**, 10.3390/ph13090222
