# Supplementary material for: Humanized monoacylglycerol acyltransferase 2 mice develop metabolic dysfunction-associated steatohepatitis
Source: J Lipid Res. 2024 Nov 5;65(12):100695. doi: 10.1016/j.jlr.2024.100695 (PMC11648239; doi:10.1016/j.jlr.2024.100695)
Supplement: Supplemental Figures and Legends [file mmc4.pdf]

Figure S1

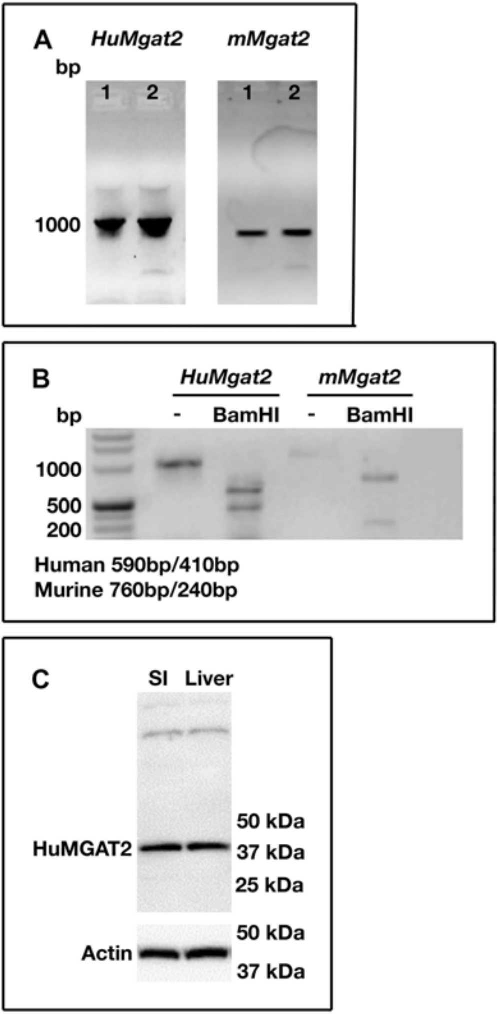
A
HuMgat2
mMgat2
bp
1
2
1
2
1000
B
HuMgat2
mMgat2
bp
-
BamHI
-
BamHI
1000
500
200
Human 590bp/410bp
Murine 760bp/240bp
C
SI
Liver
50 kDa
HuMGAT2
37 kDa
25 kDa
50 kDa
Actin
37 kDa

Figure S1. (A), cDNA fragments of *hMOGAT2* and *mMGAT2* obtained from total RNA, (B), *BamHI* restriction digestion of *hMOGAT2* and *mMGAT2* cDNAs. Digestion of *hMOGAT2* cDNA shows the correct 2 bands of 590bp and 410bp. Digestion of *mMGAT2* cDNA shows the correct 2 bands of 760bp and 240bp, (C), western blot of liver and small intestine (SI) tissues using anti-human MOGAT2 antibodies. A 38-kDa protein was detected. Actin was used as a loading control.

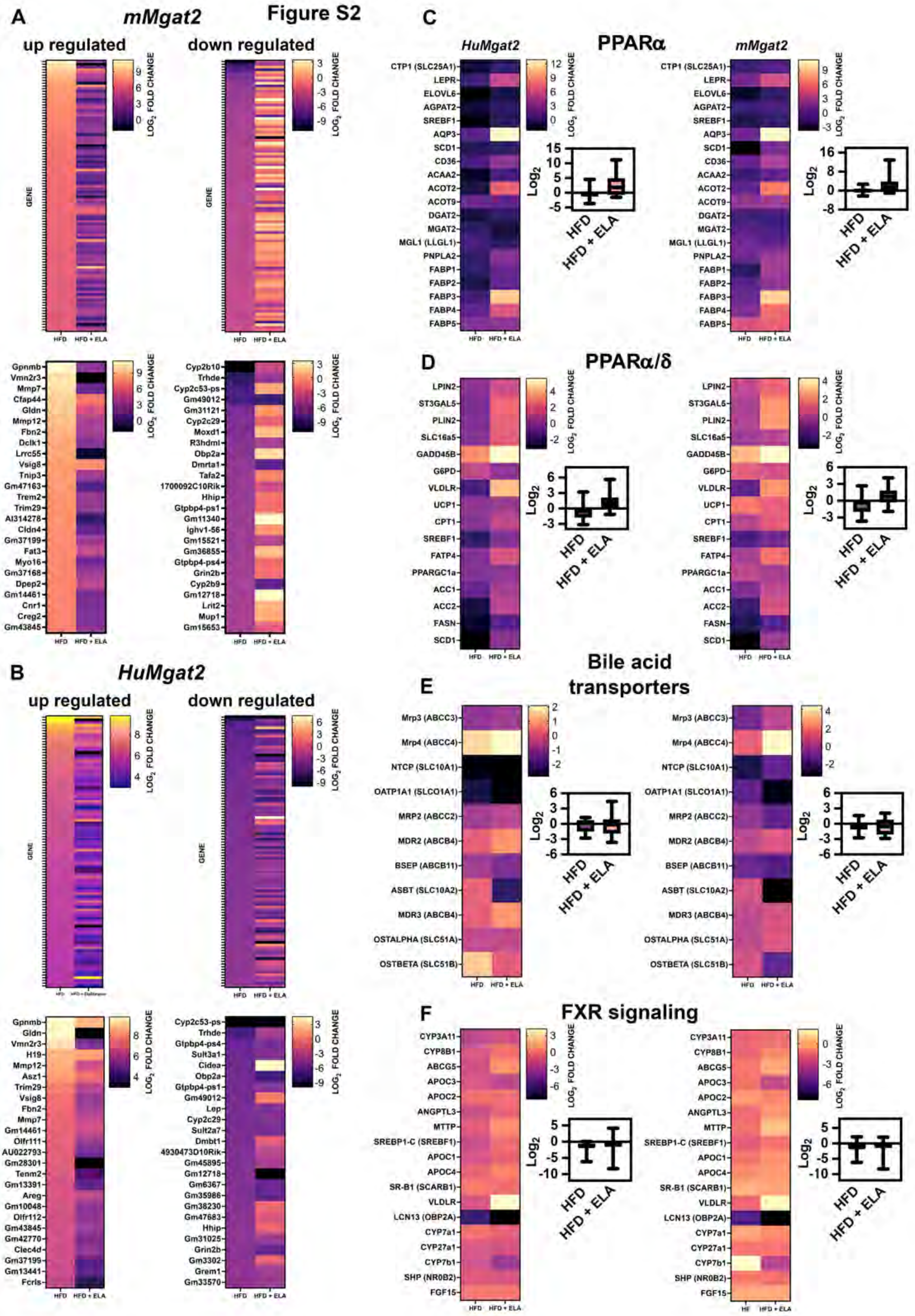
Figure S2
A
mMgat2
up regulated
down regulated
LOG2 FOLD CHANGE
GENE
HFD
HFD + ELA
Gpnmb
Vmn2r3
Mmp7
Cfap44
Gldn
Mmp12
Fbn2
Dclk1
Lrrc55
Vsig8
Tnip3
Gm47163
Trem2
Trim29
AI314278
Cldn4
Gm37199
Fat3
Myo16
Gm37168
Dpep2
Gm14461
Cnr1
Creg2
Gm43845
Cyp2b10
Trhde
Cyp2c53-ps
Gm49012
Gm31121
Cyp2c29
Moxd1
R3hdml
Obp2a
Dmrta1
Tafa2
1700092C10Rik
Hhip
Gtpbp4-ps1
Gm11340
Ighv1-56
Gm15521
Gm36855
Gtpbp4-ps4
Grin2b
Cyp2b9
Gm12718
Lrit2
Mup1
Gm15653
B
HuMgat2
up regulated
down regulated
Gpnmb
Gldn
Vmn2r3
H19
Mmp12
Asz1
Trim29
Vsig8
Fbn2
Mmp7
Gm14461
Olfr111
AU022793
Gm28301
Tenm2
Gm13391
Areg
Gm10048
Olfr112
Gm43845
Gm42770
Clec4d
Gm37199
Gm13441
Fcrls
Cyp2c53-ps
Trhde
Gtpbp4-ps4
Sult3a1
Cidea
Obp2a
Gtpbp4-ps1
Gm49012
Lep
Cyp2c29
Sult2a7
Dmbt1
4930473D10Rik
Gm45895
Gm12718
Gm6367
Gm35986
Gm38230
Gm47683
Hhip
Gm31025
Grin2b
Gm3302
Grem1
Gm33570
C
PPARα
HuMgat2
mMgat2
CTP1 (SLC25A1)
LEPR
ELOVL6
AGPAT2
SREBF1
AQP3
SCD1
CD36
ACAA2
ACOT2
ACOT9
DGAT2
MGAT2
MGL1 (LLGL1)
PNPLA2
FABP1
FABP2
FABP3
FABP4
FABP5
Log2
D
PPARα/δ
LPIN2
ST3GAL5
PLIN2
SLC16a5
GADD45B
G6PD
VLDLR
UCP1
CPT1
SREBF1
FATP4
PPARGC1a
ACC1
ACC2
FASN
SCD1
E
Bile acid transporters
Mrp3 (ABCC3)
Mrp4 (ABCC4)
NTCP (SLC10A1)
OATP1A1 (SLCO1A1)
MRP2 (ABCC2)
MDR2 (ABCB4)
BSEP (ABCB11)
ASBT (SLC10A2)
MDR3 (ABCB4)
OSTALPHA (SLC51A)
OSTBETA (SLC51B)
F
FXR signaling
CYP3A11
CYP8B1
ABCG5
APOC3
APOC2
ANGPTL3
MTTP
SREBP1-C (SREBF1)
APOC1
APOC4
SR-B1 (SCARB1)
VLDLR
LCN13 (OBP2A)
CYP7a1
CYP27a1
CYP7b1
SHP (NR0B2)
FGF15
HF

Figure S2. Heatmap analysis of RNASeq data obtained from livers of *mMgat2* and *HuMGAT2* mice. Heatmap data is the average expression values for each gene obtained from 4 individual livers. The HFD and HFD + ELA were averaged against the chow diet expression values to normalize and standardize the data for comparison. (A), top 100 and top 25 genes upregulated and down regulated in *mMgat2* mice, (B), top 100 and top 25 genes upregulated and down regulated in *HuMgat2* mice, (C), PPARα-dependent gene expression differences in livers from *mMgat2* and *HuMgat2* mice, (D), PPARβ/δ-dependent gene expression differences in livers from *mMgat2* and *HuMgat2* mice, (E), Bile acid transporter expression differences in livers from *mMgat2* and *HuMgat2* mice, (F), FXR-dependent gene expression differences in livers from *mMgat2* and *HuMgat2* mice.

Figure S3

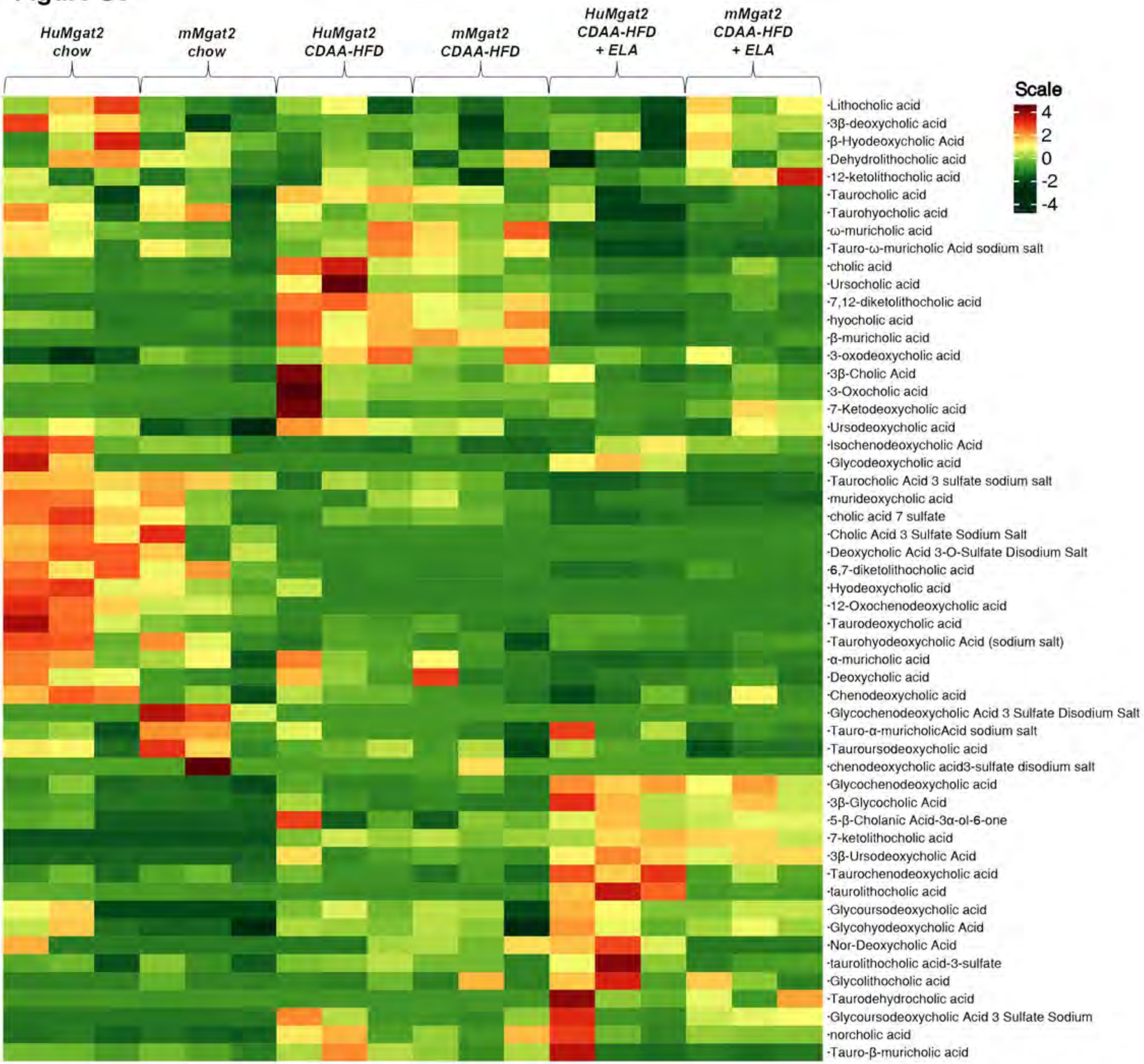
HuMgat2 chow
mMgat2 chow
HuMgat2 CDAA-HFD
mMgat2 CDAA-HFD
HuMgat2 CDAA-HFD + ELA
mMgat2 CDAA-HFD + ELA
Scale
4
2
0
-2
-4
Lithocholic acid
3β-deoxycholic acid
β-Hyodeoxycholic Acid
Dehydrolithocholic acid
12-ketolithocholic acid
Taurocholic acid
Taurohyocholic acid
ω-muricholic acid
Tauro-ω-muricholic Acid sodium salt
cholic acid
Ursocholic acid
7,12-diketolithocholic acid
hyocholic acid
β-muricholic acid
3-oxodeoxycholic acid
3β-Cholic Acid
3-Oxocholic acid
7-Ketodeoxycholic acid
Ursodeoxycholic acid
Isochenodeoxycholic Acid
Glycodeoxycholic acid
Taurocholic Acid 3 sulfate sodium salt
murideoxycholic acid
cholic acid 7 sulfate
Cholic Acid 3 Sulfate Sodium Salt
Deoxycholic Acid 3-O-Sulfate Disodium Salt
6,7-diketolithocholic acid
Hyodeoxycholic acid
12-Oxochenodeoxycholic acid
Taurodeoxycholic acid
Taurohyodeoxycholic Acid (sodium salt)
α-muricholic acid
Deoxycholic acid
Chenodeoxycholic acid
Glycochenodeoxycholic Acid 3 Sulfate Disodium Salt
Tauro-α-muricholicAcid sodium salt
Tauroursodeoxycholic acid
chenodeoxycholic acid3-sulfate disodium salt
Glycochenodeoxycholic acid
3β-Glycocholic Acid
5-β-Cholanic Acid-3α-ol-6-one
7-ketolithocholic acid
3β-Ursodeoxycholic Acid
Taurochenodeoxycholic acid
taurolithocholic acid
Glycoursodeoxycholic acid
Glycohyodeoxycholic Acid
Nor-Deoxycholic Acid
taurolithocholic acid-3-sulfate
Glycolithocholic acid
Taurodehydrocholic acid
Glycoursodeoxycholic Acid 3 Sulfate Sodium
norcholic acid
Tauro-β-muricholic acid

Figure S3. Heatmap analysis of bile acid levels in livers of *mMgat2* and *HuMGAT2* mice. Heatmap data is the average expression values for each gene obtained from 3 individual livers.

Figure S4

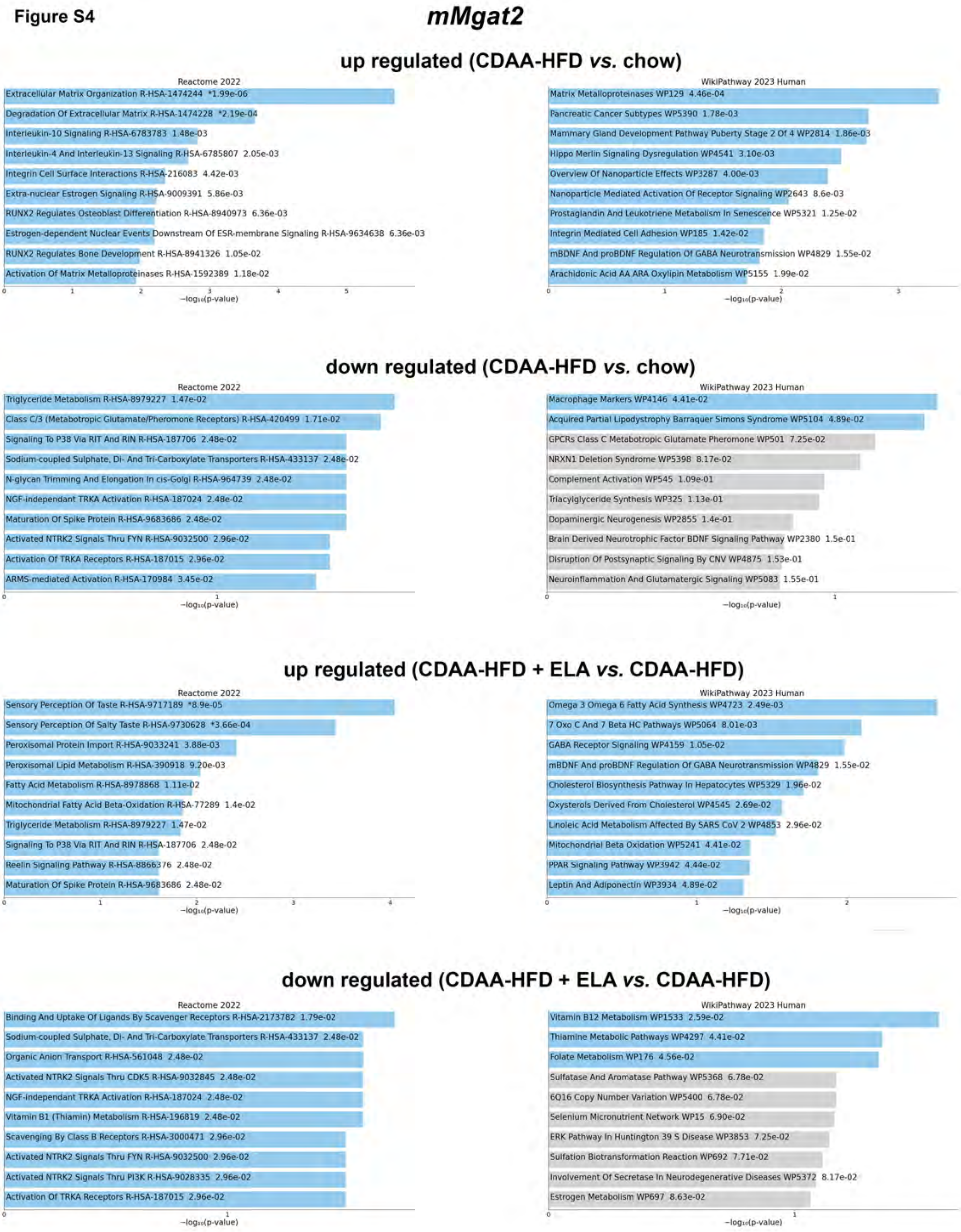
mMgat2
up regulated (CDAA-HFD vs. chow)
Reactome 2022
Extracellular Matrix Organization R-HSA-1474244 *1.99e-06
Degradation Of Extracellular Matrix R-HSA-1474228 *2.19e-04
Interleukin-10 Signaling R-HSA-6783783 1.48e-03
Interleukin-4 And Interleukin-13 Signaling R-HSA-6785807 2.05e-03
Integrin Cell Surface Interactions R-HSA-216083 4.42e-03
Extra-nuclear Estrogen Signaling R-HSA-9009391 5.86e-03
RUNX2 Regulates Osteoblast Differentiation R-HSA-8940973 6.36e-03
Estrogen-dependent Nuclear Events Downstream Of ESR-membrane Signaling R-HSA-9634638 6.36e-03
RUNX2 Regulates Bone Development R-HSA-8941326 1.05e-02
Activation Of Matrix Metalloproteinases R-HSA-1592389 1.18e-02
0 1 2 3 4 5
−log₁₀(p-value)
WikiPathway 2023 Human
Matrix Metalloproteinases WP129 4.46e-04
Pancreatic Cancer Subtypes WP5390 1.78e-03
Mammary Gland Development Pathway Puberty Stage 2 Of 4 WP2814 1.86e-03
Hippo Merlin Signaling Dysregulation WP4541 3.10e-03
Overview Of Nanoparticle Effects WP3287 4.00e-03
Nanoparticle Mediated Activation Of Receptor Signaling WP2643 8.6e-03
Prostaglandin And Leukotriene Metabolism In Senescence WP5321 1.25e-02
Integrin Mediated Cell Adhesion WP185 1.42e-02
mBDNF And proBDNF Regulation Of GABA Neurotransmission WP4829 1.55e-02
Arachidonic Acid AA ARA Oxylipin Metabolism WP5155 1.99e-02
0 1 2 3
−log₁₀(p-value)
down regulated (CDAA-HFD vs. chow)
Reactome 2022
Triglyceride Metabolism R-HSA-8979227 1.47e-02
Class C/3 (Metabotropic Glutamate/Pheromone Receptors) R-HSA-420499 1.71e-02
Signaling To P38 Via RIT And RIN R-HSA-187706 2.48e-02
Sodium-coupled Sulphate, Di- And Tri-Carboxylate Transporters R-HSA-433137 2.48e-02
N-glycan Trimming And Elongation In cis-Golgi R-HSA-964739 2.48e-02
NGF-independant TRKA Activation R-HSA-187024 2.48e-02
Maturation Of Spike Protein R-HSA-9683686 2.48e-02
Activated NTRK2 Signals Thru FYN R-HSA-9032500 2.96e-02
Activation Of TRKA Receptors R-HSA-187015 2.96e-02
ARMS-mediated Activation R-HSA-170984 3.45e-02
0 1
−log₁₀(p-value)
WikiPathway 2023 Human
Macrophage Markers WP4146 4.41e-02
Acquired Partial Lipodystrophy Barraquer Simons Syndrome WP5104 4.89e-02
GPCRs Class C Metabotropic Glutamate Pheromone WP501 7.25e-02
NRXN1 Deletion Syndrome WP5398 8.17e-02
Complement Activation WP545 1.09e-01
Triacylglyceride Synthesis WP325 1.13e-01
Dopaminergic Neurogenesis WP2855 1.4e-01
Brain Derived Neurotrophic Factor BDNF Signaling Pathway WP2380 1.5e-01
Disruption Of Postsynaptic Signaling By CNV WP4875 1.53e-01
Neuroinflammation And Glutamatergic Signaling WP5083 1.55e-01
0 1
−log₁₀(p-value)
up regulated (CDAA-HFD + ELA vs. CDAA-HFD)
Reactome 2022
Sensory Perception Of Taste R-HSA-9717189 *8.9e-05
Sensory Perception Of Salty Taste R-HSA-9730628 *3.66e-04
Peroxisomal Protein Import R-HSA-9033241 3.88e-03
Peroxisomal Lipid Metabolism R-HSA-390918 9.20e-03
Fatty Acid Metabolism R-HSA-8978868 1.11e-02
Mitochondrial Fatty Acid Beta-Oxidation R-HSA-77289 1.4e-02
Triglyceride Metabolism R-HSA-8979227 1.47e-02
Signaling To P38 Via RIT And RIN R-HSA-187706 2.48e-02
Reelin Signaling Pathway R-HSA-8866376 2.48e-02
Maturation Of Spike Protein R-HSA-9683686 2.48e-02
0 1 2 3 4
−log₁₀(p-value)
WikiPathway 2023 Human
Omega 3 Omega 6 Fatty Acid Synthesis WP4723 2.49e-03
7 Oxo C And 7 Beta HC Pathways WP5064 8.01e-03
GABA Receptor Signaling WP4159 1.05e-02
mBDNF And proBDNF Regulation Of GABA Neurotransmission WP4829 1.55e-02
Cholesterol Biosynthesis Pathway In Hepatocytes WP5329 1.96e-02
Oxysterols Derived From Cholesterol WP4545 2.69e-02
Linoleic Acid Metabolism Affected By SARS CoV 2 WP4853 2.96e-02
Mitochondrial Beta Oxidation WP5241 4.41e-02
PPAR Signaling Pathway WP3942 4.44e-02
Leptin And Adiponectin WP3934 4.89e-02
0 1 2
−log₁₀(p-value)
down regulated (CDAA-HFD + ELA vs. CDAA-HFD)
Reactome 2022
Binding And Uptake Of Ligands By Scavenger Receptors R-HSA-2173782 1.79e-02
Sodium-coupled Sulphate, Di- And Tri-Carboxylate Transporters R-HSA-433137 2.48e-02
Organic Anion Transport R-HSA-561048 2.48e-02
Activated NTRK2 Signals Thru CDK5 R-HSA-9032845 2.48e-02
NGF-independant TRKA Activation R-HSA-187024 2.48e-02
Vitamin B1 (Thiamin) Metabolism R-HSA-196819 2.48e-02
Scavenging By Class B Receptors R-HSA-3000471 2.96e-02
Activated NTRK2 Signals Thru FYN R-HSA-9032500 2.96e-02
Activated NTRK2 Signals Thru PI3K R-HSA-9028335 2.96e-02
Activation Of TRKA Receptors R-HSA-187015 2.96e-02
0 1
−log₁₀(p-value)
WikiPathway 2023 Human
Vitamin B12 Metabolism WP1533 2.59e-02
Thiamine Metabolic Pathways WP4297 4.41e-02
Folate Metabolism WP176 4.56e-02
Sulfatase And Aromatase Pathway WP5368 6.78e-02
6Q16 Copy Number Variation WP5400 6.78e-02
Selenium Micronutrient Network WP15 6.90e-02
ERK Pathway In Huntington 39 S Disease WP3853 7.25e-02
Sulfation Biotransformation Reaction WP692 7.71e-02
Involvement Of Secretase In Neurodegenerative Diseases WP5372 8.17e-02
Estrogen Metabolism WP697 8.63e-02
0 1
−log₁₀(p-value)

Figure S4. Liver bioinformatics pathway analysis livers of *mMGAT2* mice. Wikipathway 2023 human and Reactome 2022 analysis was performed on the top 100 statistically significant genes that were up regulated or down regulated in *mMgat2* mice. The diet conditions compared are indicated.

Figure S5

HuMgat2

up regulated (CDAA-HFD *vs.* chow)

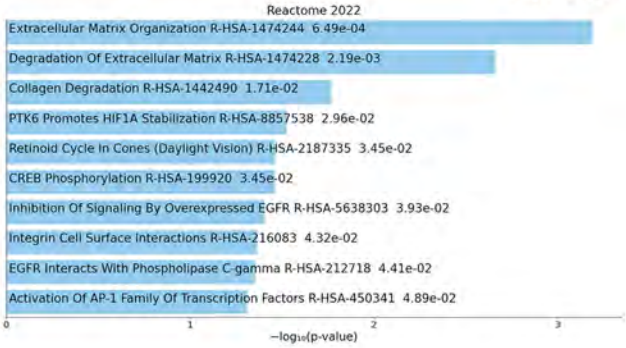

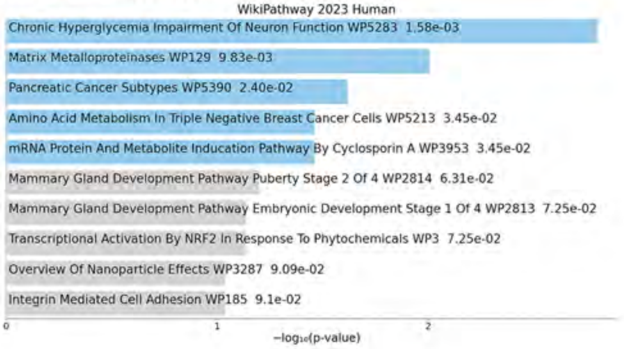

down regulated (CDAA-HFD *vs.* chow)

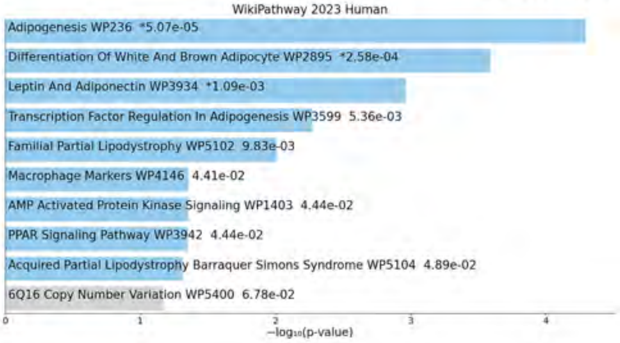

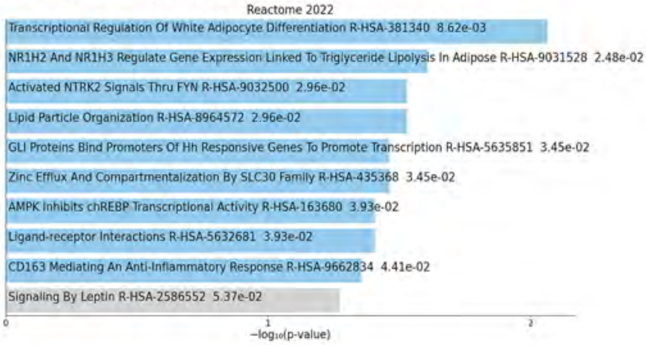

up regulated (CDAA-HFD + ELA *vs.* CDAA-HFD)

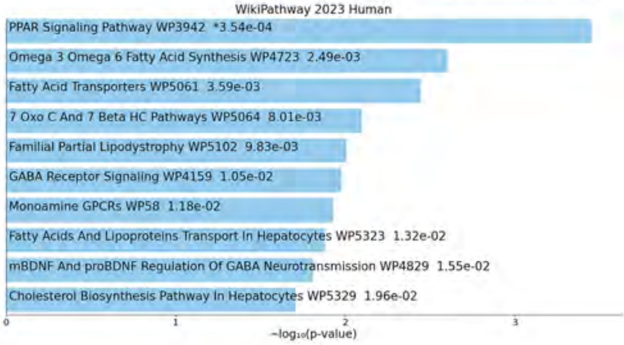

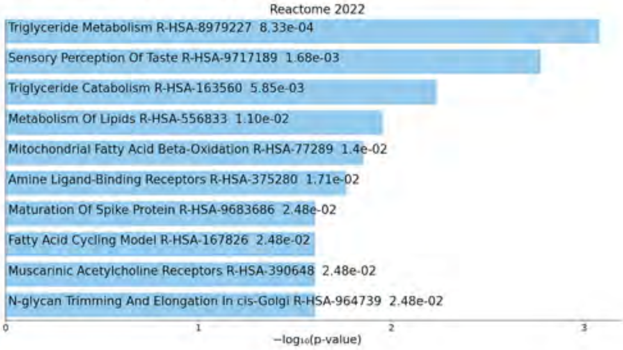

down regulated (CDAA-HFD + ELA *vs.* CDAA-HFD)

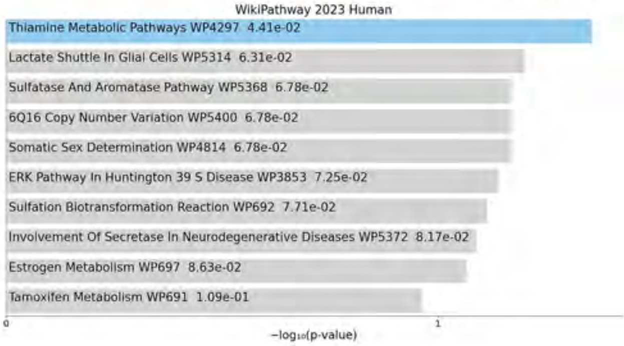

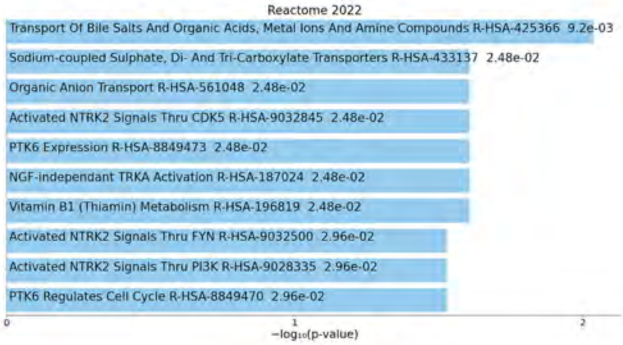

Figure S5. Liver bioinformatics pathway analysis livers of *huMGAT2* mice. Wikipathway 2023 human and Reactome 2022 analysis was performed on the top 100 statistically significant genes that were up regulated or down regulated in *huMgat2* mice. The diet conditions compared are indicated.
