# Supplementary material for: Humanized monoacylglycerol acyltransferase 2 mice develop metabolic dysfunction-associated steatohepatitis
Source: J Lipid Res. 2024 Nov 5;65(12):100695. doi: 10.1016/j.jlr.2024.100695 (PMC11648239; doi:10.1016/j.jlr.2024.100695)

Mouse Mogat2 Humanization Project

Objective:

- To create a mouse Mogat2 humanized model in C57BL/6 mice.

Gene and protein information:

Mogat2 monoacylglycerol O-acyltransferase 2 [*Mus musculus* (house mouse)]

Gene ID: 233549, updated on 4-Mar-2017

Summary

**Official Symbol**	Mogat2 provided by MGI
**Official Full Name**	monoacylglycerol O-acyltransferase 2 provided by MGI
**Primary source**	MGI:MGI:2663253
**See related**	Ensembl:ENSMUSG00000052396 Vega:OTTMUSG00000027299
**Gene type**	protein coding
**RefSeq status**	VALIDATED
**Organism**	Mus musculus
**Lineage**	Eukaryota; Metazoa; Chordata; Craniata; Vertebrata; Euteleostomi; Mammalia; Eutheria; Euarchontoglires; Glires; Rodentia; Myomorpha; Muroidea; Muridae; Murinae; Mus; Mus
**Also known as**	MGAT2; Mgat1l; DGAT2L5
**Orthologs**	human all

Genomic context

Location: 7; 7 E1

See Mogat2 in Genome Data Viewer Map Viewer

Exon count: 7

Annotation release	Status	Assembly	Chr	Location
106	current	GRCm38.p4 (GCF_000001635.24)	7	NC_000073.6 (99219084..99238611, complement)
Build 37.2	previous assembly	MGSCv37 (GCF_000001635.18)	7	NC_000073.5 (106367594..106387121, complement)

Chromosome 7 - NC_000073.6

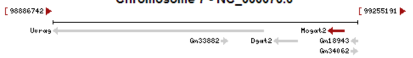

MOGAT2 monoacylglycerol O-acyltransferase 2 [*Homo sapiens* (human)]

Gene ID: 80168, updated on 5-Mar-2017

Summary

**Official Symbol**	MOGAT2 provided by HGNC
**Official Full Name**	monoacylglycerol O-acyltransferase 2 provided by HGNC
**Primary source**	HGNC:HGNC:23248
**See related**	Ensembl:ENSG00000166391 MIM:610270; Vega:OTTHUMG00000165341
**Gene type**	protein coding
**RefSeq status**	REVIEWED
**Organism**	Homo sapiens
**Lineage**	Eukaryota; Metazoa; Chordata; Craniata; Vertebrata; Euteleostomi; Mammalia; Eutheria; Euarchontoglires; Primates; Haplorrhini; Catarrhini; Hominidae; Homo
**Also known as**	hDC5; MGAT2; DGAT2L5; DGAT2L5.
**Summary**	The protein encoded by this gene is an enzyme that catalyzes the synthesis of diacylglycerol from 2-monoacylglycerol and fatty acyl-CoA. The encoded protein is important in the uptake of dietary fat by the small intestine. This protein forms a complex with diacylglycerol O-acyltransferase 2 in the endoplasmic reticulum, and this complex catalyzes the synthesis of triacylglycerol. [provided by RefSeq, Dec 2015]
**Orthologs**	mouse all

Genomic context

Location: 11q13.5

See MOGAT2 in Genome Data Viewer Map Viewer

Exon count: 7

Annotation release	Status	Assembly	Chr	Location
108	current	GRCh38.p7 (GCF_000001405.33)	11	NC_000011.10 (75717819..75732958)
105	previous assembly	GRCh37.p13 (GCF_000001405.25)	11	NC_000011.9 (75428934..75442331)

Chromosome 11 - NC_000011.10

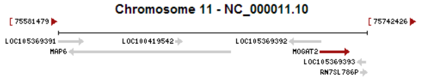

Transcripts:

1. Mouse Mogat2 gene:

This gene has 2 transcripts.

Gene: Mogat2 ENSMUSG00000052396

**Description**	monoacylglycerol O-acyltransferase 2 [Source:MGI Symbol;Acc:MGI:2663253]
**Synonyms**	Mgat1l, Mgat2, MGAT2, DGAT2L5, Dgat2l5
**Location**	Chromosome 7: 99,219,084-99,238,619 reverse strand.<br>GRCm38:CM001000.2
**About this gene**	This gene has 2 transcripts (splice variants), 93 orthologues, 5 paralogues, is a member of 1 Ensembl protein family and is associated with 14 phenotypes.
**Transcripts**	Hide transcript table

Show/hide columns (1 hidden) Filter

Name	Transcript ID	bp	Protein	Biotype	CCDS	UniProt	RefSeq	Flags
Mogat2-001	ENSMUST00000064231.7	1780	334aa	Protein coding	CCDS21478	Q80W94	NM_177448<br>NP_803231	TSL:1 GENCODE basic APPRIS P1
Mogat2-002	ENSMUST00000132343.1	354	No protein	Processed transcript	-	-	-	TSL:3

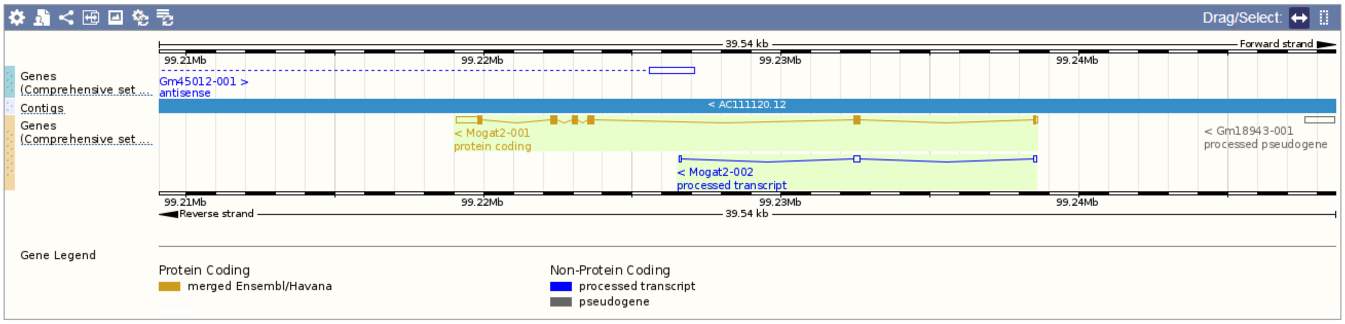

2. Human MOGAT2 gene:

This gene has 4 transcripts.

Gene: MOGAT2 ENSG00000166391

**Description**	monoacylglycerol O-acyltransferase 2 [Source:HGNC Symbol;Acc:HGNC:23248]
**Synonyms**	MGAT2, hDC5, DGAT2L5., DGAT2L5, FLJ22644, DC5
**Location**	Chromosome 11: 75,717,819-75,732,958 forward strand.<br>GRCh38:CM000673.2
**About this gene**	This gene has 4 transcripts (splice variants), 93 orthologues, 6 paralogues, is a member of 1 Ensembl protein family and is associated with 1 phenotype.
**Transcripts**	Hide transcript table

Show/hide columns (1 hidden)

Name	Transcript ID	bp	Protein	Biotype	CCDS	UniProt	RefSeq	Flags
**MOGAT2-001**	ENST00000198801.9	1404	334aa	Protein coding	CCDS8240	Q3SYC2	NM_025098<br>NP_079374	TSL:1 GENCODE basic APPRIS P1
**MOGAT2-004**	ENST00000526712.1	3204	252aa	Protein coding	-	Q3SYC2	-	TSL:2 GENCODE basic
**MOGAT2-003**	ENST00000525093.5	1650	284aa	Nonsense mediated decay	-	Q3SYC2	-	TSL:2
**MOGAT2-005**	ENST00000624180.1	1956	No protein	TEC	-	-	-	TSL:NA

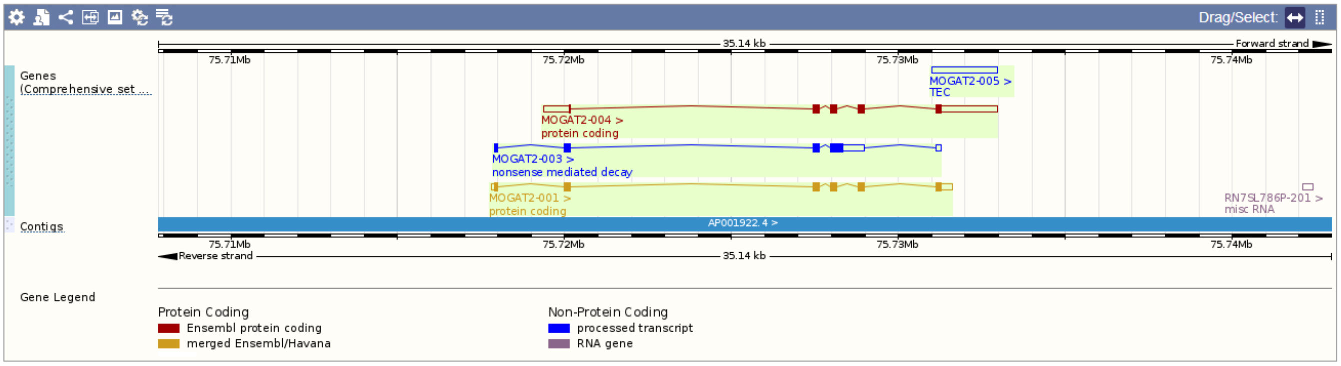

Summary:

1. The Mogat2 gene (NCBI Reference Sequence: NM_177448.4) is located on mouse chromosome 7. Six exons have been identified, with the ATG start codon in exon 1 and the TAA stop codon in exon 6.
2. The MOGAT2 gene (NCBI Reference Sequence: NM_025098.3) is located on human chromosome 11. Six exons have been identified, with the ATG start codon in exon 1 and the TGA stop codon in exon 6.
3. For the KI model, the sequences from ATG start codon in exon 1 to TAA stop codon in exon 6 of the endogenous mouse Mogat2 gene will be replaced with CDS of the human MOGAT2 gene.
4. To engineer the targeting vector, homology arms will be generated by PCR using BAC clone RP23-363C16 or RP23-111F4 from the C57BL/6J library as template.
5. In the targeting vector, the Neo cassette will be flanked by LoxP sites. DTA will be used for negative selection.
6. The constitutive KI allele will be obtained after Cre-mediated recombination.
7. C57BL/6 ES cells will be used for gene targeting.

Template Needed	Cost of Purchase
BAC: RP23-363C16	TBD
BAC: RP23-111F4	TBD
CDS of the human MOGAT2 gene: NM_025098.3	$ 362

CDS of the human MOGAT2 gene:

```
  1 ATGGTA GAGTTC GCGCCC TTGTTT ATGCCG TGGGAG CGCAGG CTGCAG ACACTT GCTGTC
 61 CTACAG TTTGTC TTCTCC TTCTTG GCACTG GCCGAG ATCTGC ACTGTG GGCTTC ATAGCC
121 CTCCTG TTTACA AGATTC TGGCTC CTCACT GTCCTG TATGCG GCCTGG TGGTAT CTGGAC
181 CGAGAC AAGCCA CGGCAG GGGGGC CGGCAC ATCCAG GCCATC AGGTGC TGGACT ATATGG
241 AAGTAC ATGAAG GACTAT TTCCCC ATCTCG CTGGTC AAGACT GCTGAG CTGGAC CCCTCT
301 CGGAAC TACATT GCGGGC TTCCAC CCCCAT GGAGTC CTGGCA GTCGGA GCCTTT GCCAAC
361 CTGTGC ACTGAG AGCACA GGCTTC TCTTCG ATCTTC CCCGGT ATCCGC CCCCAT CTGATG
421 ATGCTG ACCTTG TGGTTC CGGGCC CCCTTC TTCAGA GATTAC ATCATG TCTGCA GGGTTG
481 GTCACA TCAGAA AAGGAG AGTGCT GCTCAC ATTCTG AACAGG AAGGGT GGCGGA AACTTG
541 CTGGGC ATCATT GTAGGG GGTGCC CAGGAG GCCCTG GATGCC AGGCCT GGATCC TTCACG
601 CTGTTA CTGCGG AACCGA AAGGGC TTCGTC AGGCTC GCCCTG ACACAC GGGGCA CCCCTG
```

661 GTGCCA ATCTTC TCCTTC GGGGAG AATGAC CTATTT GACCAG ATTCCC AACTCT TCTGGC
721 TCCTGG TTACGC TATATC CAGAAT CGGTTG CAGAAG ATCATG GGCATC TCCCTC CCACTC
781 TTTCAT GGCCGT GGTGTC TTCCAG TACAGC TTTGGT TTAATA CCCTAC CGCCGG CCCATC
841 ACCACT GTGGTG GGGAAG CCCATC GAGGTA CAGAAG ACGCTG CATCCC TCGGAG GAGGAG
901 GTGAAC CAGCTG CACCAG CGTTAT ATCAAA GAGCTG TGCAAC CTCTTC GAGGCC CACAAA
961 CTTAAG TTCAAC ATCCCT GCTGAC CAGCAC TTGGAG TTCTGC TGA

Overview of the Targeting Strategy

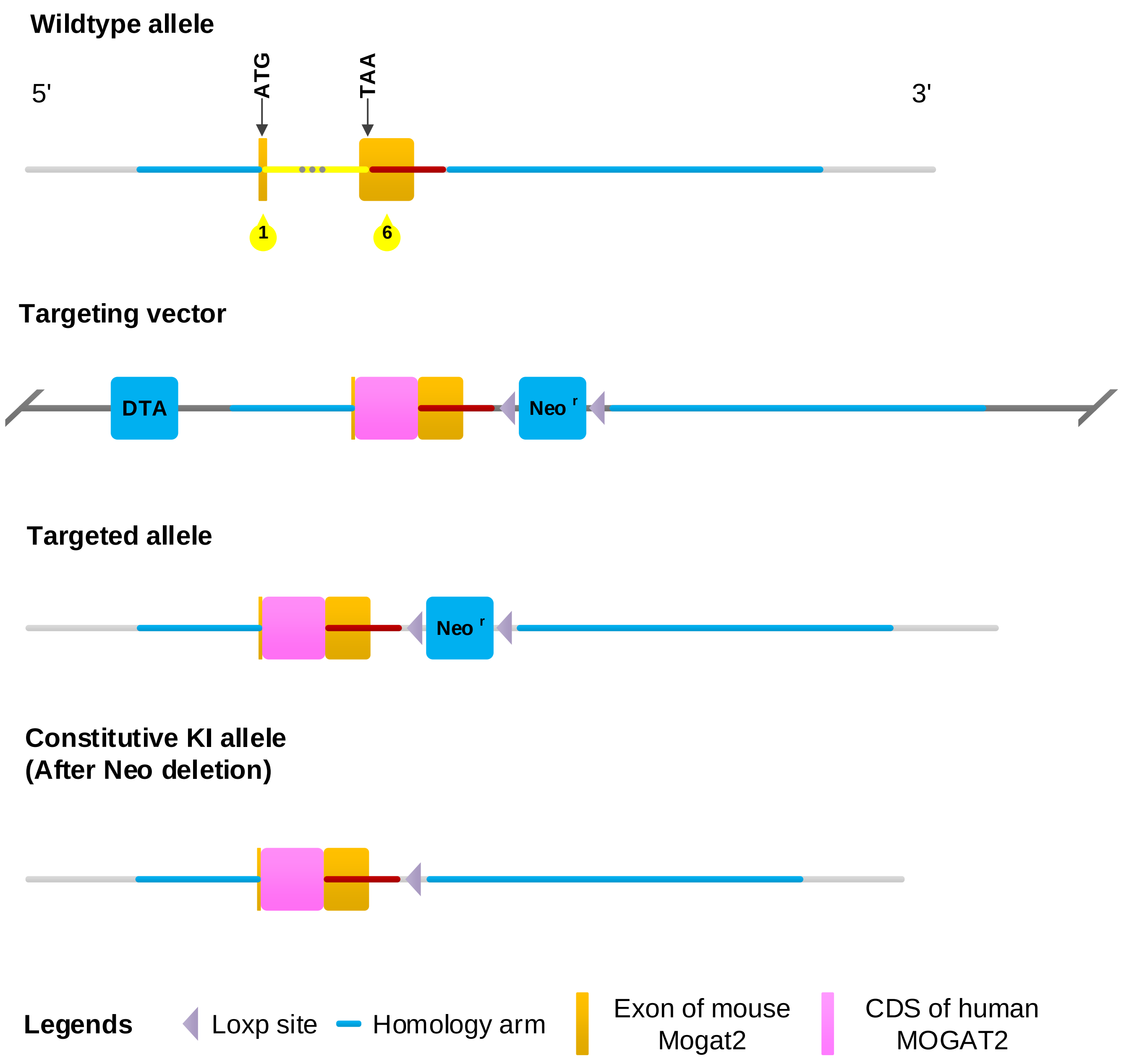

Supplement: Cyagen HuMgat2 Project 12.2023 [file mmc5.pdf]
